# Quantitative proteomic screen identifies annexin A2 as a host target for *Salmonella* pathogenicity island-2 effectors SopD2 and PipB2

**DOI:** 10.1038/s41598-021-02795-x

**Published:** 2021-12-08

**Authors:** Katelyn Knuff-Janzen, Antonio Serapio-Palacios, James McCoy, Zakhar Krekhno, Kyung-Mee Moon, Wanyin Deng, Leonard J. Foster, B. Brett Finlay

**Affiliations:** 1 Michael Smith Laboratories, University of British Columbia, Vancouver, BC Canada; 2 Department of Microbiology and Immunology, University of British Columbia, Vancouver, BC Canada; 3 Department of Biochemistry and Molecular Biology, University of British Columbia, Vancouver, BC Canada

**Keywords:** Pathogens, Proteomics

## Abstract

Intracellular pathogens need to establish an intracellular replicative niche to promote survival and replication within the hostile environment inside the host cell. *Salmonella enterica* serovar Typhimurium (*S.* Typhimurium) initiates formation of the unique *Salmonella*-containing vacuole and an extensive network of *Salmonella-*induced tubules in order to survive and thrive within host cells. At least six effectors secreted by the type III secretion system encoded within *Salmonella* pathogenicity island-2 (SPI-2), namely SifA, SopD2, PipB2, SteA, SseJ, and SseF, purportedly manipulate host cell intracellular trafficking and establish the intracellular replicative niche for *S.* Typhimurium. The phenotypes of these effectors are both subtle and complex, complicating elucidation of the mechanism underpinning host cell manipulation by *S.* Typhimurium. In this work we used stable isotope labeling of amino acids in cell culture (SILAC) and a *S.* Typhimurium mutant that secretes increased amounts of effectors to identify cognate effector binding partners during infection. Using this method, we identified the host protein annexin A2 (AnxA2) as a binding partner for both SopD2 and PipB2 and were able to confirm its binding to SopD2 and PipB2 by reciprocal pull down, although there was a low level of non-specific binding of SopD2-2HA and PipB2-2HA to the Ni-Sepharose beads present. We further showed that knockdown of AnxA2 altered the intracellular positioning of the *Salmonella* containing vacuole (SCV). This suggests that AnxA2 plays a role in the subcellular positioning of the SCV which could potentially be mediated through protein–protein interactions with either SopD2 or PipB2. This demonstrates the value of studying effector interactions using proteomic techniques and natural effector delivery during infection rather than transfection.

## Introduction

*Salmonella enterica* is a facultative intracellular pathogen capable of causing disease in humans and a broad range of animal hosts. The medically relevant *Salmonella enterica* serovars are among the leading causes of gastroenteritis and bacteremia worldwide^1^. The pathogenesis of *Salmonella enterica* serovar Typhimurium (*S.* Typhimurium) is largely mediated by two distinct type III secretion systems encoded on *Salmonella* pathogenicity islands 1 and 2 (T3SS1 and T3SS2, respectively)^2^. These type III secretion systems transport bacterial effector proteins into the cytoplasm of the host cell where they specifically target host processes to promote both invasion and intracellular survival of *S.* Typhimurium^3^. Effectors secreted by the T3SS1 largely function to promote invasion into epithelial cells, whereas effectors secreted by the T3SS2 promote intracellular survival and replication within both epithelial and phagocytic cells^4^.

Critical to the success of *S.* Typhimurium as an intracellular pathogen is establishing a unique intracellular replicative niche. A subset of secreted effectors contribute to formation of the *Salmonella* containing vacuole (SCV) to protect intracellular *S.* Typhimurium from host defenses^4^. At 4–6 h post invasion, *S.* Typhimurium induces formation of filamentous structures known as *Salmonella*-induced filaments (SIFs) that radiate outwards from the SCV along microtubules^5–9^. The two interrelated processes of SIF biogenesis and SCV membrane maintenance are in large part resultant from controlled interactions with the host endomembrane system mediated by the T3SS2-secreted effectors SifA, SopD2, SteA, PipB2, SseJ, SseF, and SseG^10^.

We have previously shown that effector phenotypes during infection are nuanced and complex in ways that are not captured by single effector studies as it is likely that effectors work in concert to mediate multiple aspects of SIF biogenesis or SCV membrane maintenance^11^. Host binding partners for SifA, SopD2, SteA, PipB2, SseJ, SseF, and SseG have previously been identified largely through single-effector transfection studies (reviewed in Ref.^10^). Such studies express a single effector out of the context of a native infection, and therefore would not be expected to demonstrate a phenotype if multiple T3SS2 effectors cooperate to form SIFs or maintain the SCV membrane. The overlapping and redundant nature of many T3SS2 effectors further hampers efforts to discover novel effector functions. Characterization of the protein–protein interactions mediating these critical infection stages is key to understanding the significance of T3SS2-secreted effectors during *S.* Typhimurium infection.

We employed SILAC (stable isotope labelling with amino acids in cell culture) to identify cognate binding partners of the effectors associated with SIF biogenesis, SCV membrane maintenance, and intracellular positioning of the SCV during infection. SILAC is a quantitative mass spectrometry-based technique that relies on non-radioactive isotopic labelling of particular amino acids to detect differences in protein abundances in different samples allowing identification of specific and non-specific protein–protein interactions^12^. This methodology permits identification and quantification of effector binding partners, whether that be a host protein or a secreted bacterial effector. Our lab previously used the SILAC method to identify protein–protein interactions during *S.* Typhimurium infection by incorporating stable isotope-labelled amino acids into the entire proteome of host cells, followed by effector transfection, immunoprecipitation of effector complexes, and analysis by mass spectrometry^13^.

We hypothesize that the nuanced phenotypes resulting from effector deletion studies arise from effectors targeting the same host protein or forming an effector-effector complex. To investigate this, we examined a series of T3SS2-secreted effector protein–protein interactions during infection by performing a series of immunoprecipitation experiments in SILAC-labelled host cells infected with *S.* Typhimurium strains secreting increased abundance of HA-tagged T3SS2-effectors through overexpression of the T3SS2 transcriptional regulator SsrB. Using this approach, we demonstrate the utility of examining the role of multiple effectors during infection and find a common host binding partner for two T3SS2 effectors.

## Materials and methods

### Bacterial strains, culture conditions, and plasmids

Bacterial strains used in this work are described in Table 1. All strains were routinely grown in Lysogeny Broth (LB) medium at 37 °C with shaking. For growth of the *E. coli* MFD*pir* strain, media were supplemented with dl-2,6-Diaminopimelic acid (DAP) at a final concentration of 0.3 mM when appropriate. Antibiotics were used at the following concentration when required: streptomycin 50 µg/mL, chloramphenicol 30 µg/mL.

**Table 1:** Bacterial strains used in this study.

Strain designation	Relevant characteristics/genotype	Source/referenced
SL1344	*S.* Typhimurium wild type strain, *hisG*	^14^
*E. coli* MC1061*λpir*	*hsdR mcrB araD139 ∆(araABC-leu)7679 ∆lacX74 gal1 galK rpsL thi* λ*pir*	^15^
*E. coli* MFD*pir*	*MG1655 RP4-2-TC::[∆Mu1::aac(3)IV-∆aphA-∆nic35-∆Mu2::zeo] ∆dapA::(erm-pir) ∆recA*	^16^
*E. coli* DH10B	F^-^ *araDJ39 ∆(ara, leu)7697 ∆lacX74 galU galK rpsL deoR ɸ80dlacZ∆M15 endAI nupG recAl mcrA ∆(mrr hsdRMS mcrBC*)	^17^
*∆invA*	*invA* single deletion mutant in SL1344 genetic background; non-functional T3SS1 apparatus	^18^
*∆ssaR*	*ssaR* single deletion mutant in SL1344 genetic background; non-functional T3SS2 apparatus	^19^
T3SS2^+^	chromosomal duplication of *ssrB* in SL1344 resulting in increased expression of SPI-2 and its associated effectors	This study
T3SS2^+^ *∆sifA*	*sifA* single deletion mutant in the *ssrB* overexpressing mutant background	This study
T3SS2^+^ *∆sifA*-pSifA-2HA	*sifA* single deletion mutant in the *ssrB* overexpressing mutant background complemented with pSifA-2HA	This study
T3SS2^+^ *∆sopD2*	*sopD2* single deletion mutant in the *ssrB* overexpressing mutant background	This study
T3SS2^+^ *∆sopD2*-pSopD2-2HA	*sopD2* single deletion mutant in the *ssrB* overexpressing mutant background complemented with pSopD2-2HA	This study
T3SS2^+^ *∆pipB2*	*pipB2* single deletion mutant in the *ssrB* overexpressing mutant background	This study
T3SS2^+^ *∆pipB2*-pPipB2-2HA	*pipB2* single deletion mutant in the *ssrB* overexpressing mutant background complemented with pPipB2-2HA	This study
T3SS2^+^ *∆steA*	*steA* single deletion mutant in the *ssrB* overexpressing mutant background	This study
T3SS2^+^ *∆steA*-pSteA-2HA	*steA* single deletion mutant in the *ssrB* overexpressing mutant background complemented with pSteA-2HA	This study
T3SS2^+^ *∆sseJ*	*sseJ* single deletion mutant in the *ssrB* overexpressing mutant background	This study
T3SS2^+^ *∆sseJ*-pSseJ-2HA	*sseJ* single deletion mutant in the *ssrB* overexpressing mutant background complemented with pSseJ-2HA	This study
T3SS2^+^ *∆sseF*	*sseF* single deletion mutant in the *ssrB* overexpressing mutant background	This study
T3SS2^+^ *∆sseF*-pSseF-2HA	*sseF* single deletion mutant in the *ssrB* overexpressing mutant background complemented with pSseF-2HA	This study
T3SS2^+^ pACYC-2HA	*ssrB* overexpressing mutant transformed with pACYC184 containing a 2xHA tag under the control of the native promoter for *pipB2* expression	This study

Unless otherwise indicated, strains are isogenic mutants of *S.* Typhimurium SL1344.

### Plasmid construction

Plasmids constructed and used in this study are listed in Table 2. Plasmids pSopD2-2HA and pSifA-2HA were made previously^20,21^ as were plasmids pRE112*∆sifA*, pRE112*∆sopD2*, pRE112*∆pipB2*, pRE112*∆steA*, pRE112*∆sseJ*, and pRE112*∆sseF*^11^. Complementation vectors and gene deletion/insertion vectors were routinely maintained in *E. coli* DH10B and MC1061λ*pir*, respectively. Primers are listed in Table 3.

**Table 2:** Plasmids used in this study.

Plasmid designation	Relevant characteristics/genotype	Source/references
pRE112-*ssrB*	pRE112 containing *ssrB* and homologous regions for insertion	This study
pRE112*ΔsifA*	pRE112 containing homologous regions surrounding *sifA* for chromosomal *sifA* deletion	^11^
pRE112*ΔsopD2*	pRE112 containing homologous regions surrounding *sopD2* for chromosomal *sopD2* deletion	^11^
pRE112*ΔpipB2*	pRE112 containing homologous regions surrounding *pipB2* for chromosomal *pipB2* deletion	^11^
pRE112*ΔsteA*	pRE112 containing homologous regions surrounding *steA* for chromosomal *steA* deletion	^11^
pRE112*ΔsseJ*	pRE112 containing homologous regions surrounding *sseJ* for chromosomal *sseJ* deletion	^11^
pRE112Δ*sseF*	pRE112 containing homologous regions surrounding *sseF* for chromosomal *sseF* deletion	^11^
pSifA-2HA	Tandem HA Tag in the middle of SifA in pACYC184	^20^
pSopD2-2HA	SopD2 with a tandem C-terminal HA tag in pACYC184	^21^
pPipB2-2HA	PipB2 with a tandem C-terminal HA tag in pACYC184	This study
pSteA-2HA	SteA with a tandem C-terminal HA tag in pACYC184	This study
pSseJ-2HA	SseJ with a tandem C-terminal HA tag in pACYC184	This study
pSseF-2HA	SseF with a tandem C-terminal HA tag in pACYC184	This study
pACYC-2HA	Tandem HA Tag under the control of the native *pipB2* promoter in pACYC184	This study

**Table 3:** Primers used in this study.

Primer name	5ʹ to 3ʹ sequence	Description
KK_001	GAGCTCTCCCGGGAATTCATGCAGTTCAC	Linearization of pRE112 maintaining SacI and **KpnI** cut sites
KK_002	**GGTACC**TCTAGAAGAAGCTTGGGA	
KK_003	CAAGCTTCTTCTAGAGGTACCCCGTCTTCGCTGATATCCCAC	Amplification of region upstream of *ssrB* insertion
KK_004	CTCAGATAATCAACATATCGAAAGAAATTTTTC	
KK_005	CGATATGTTGATTATCTGAGCAGATGATATGGTCATTAATAGCAAG	Amplification of *ssrB* and native promoter
KK_006	GCGTTAGTGGTATTAATCGTTAATACTCTATTAACCTCATTCTTCGGGC	
KK_007	GAGGTTAATAGAGTATTAACGATTAATACCACTAACGCTAAAACGCAC	Amplification of region downstream of *ssrB* insertion
KK_008	ATGAATTCCCGGGAGAGCTCGGTGATGCGGTAATGTCGCTGC	
KK_009	GTACGCGTCGACCCGAGACGGTAGCCTGATTGAGTTAAACG	Amplification of *pipB2* plus native promoter, SalI and **XhoI** cut sites
KK_010	GTACGC**CTCGAG**AATATTTTCACTATAAAATTCGTTAAAGAGTGTTTGTGTGC	
KK_011	GTACGCGTCGACGCGCTTCCCCATCCCAAACCACC	Amplification of *sseJ* plus native promoter, SalI and **XhoI** cut sites
KK_012	GTACGC**CTCGAG**TTCAGTGGAATAATGATGAGCTATAAAACTTTCTAACATTATGGC	
KK_013	GTACGCGTCGACGCGACGGGCGCTCACCAATC	Amplification of *steA* plus native promoter, SalI and **XhoI** cut sites
KK_014	GTACGC**CTCGAG**ATAATTGTCCAAATAGTTATGGTAGCGAGCTTTTATGTCGG	
KK_015	GTACGCGTCGACGAAGAGAACAACGGCAAGTTACAGGATCCGC	Amplification of *sseABCDEFG* operon promoter, SalI cut site
KK_016	GATTGTTATTTTCACGTGCCCCTCCATATACACGATAGATAATTAACGTGCTAAC	
KK_017	CTATCGTGTATATGGAGGGGCACGTGAAAATAACAATCAATAGGTATGATGATGAAAG	Amplification of *sscB* chaperone and *sseF*
KK_018	GTACGC**CTCGAG**TGGTTCTCCCCGAGATGTATGATCAG	Reverse primer﻿ to amplify *sseF* and *sscB*, **XhoI** cut site
KK_019	GTACGC**CTCGAG**GAGTGAACGCTCCATATATTTTCTCCCAGAGACAG	Amplification and linearization of pPipB2-2HA, **XhoI** cut site
KK_020	CGAATTTTATAGTGAAAATATT**CTCGAG**TATCCGTATG	

Bold and underline indicate restriction endonuclease cut sites.

The plasmid pRE112-*ssrB* was constructed using primers KK_001 and KK_002 to amplify linear pRE112 from its KpnI to SacI unique restriction sites. For PCR, Phusion High-Fidelity DNA Polymerase (NEB) was used. Linearized pRE112 plasmid backbone was subsequently digested with DpnI (NEB) to remove any remaining circular template DNA. To generate an unmarked gene insertion, the region upstream of the *ssrB* insertion site was amplified using primers KK_003 and KK_004 and the region downstream of the insertion site was amplified using primers KK_007 and KK_008. The *ssrB* coding region and native promoter were amplified using primers KK_005 and KK_006. Linearized pRE112, upstream insertion site fragment, *ssrB* fragment, and downstream insertion site fragment were assembled to form pRE112-*ssrB* using Gibson Assembly^22^.

Protein coding and regulatory regions of *pipB2, steA, sseJ,* and *sseF* were amplified from SL1344 genomic DNA using the indicated primers: *pipB2*: KK_009 and KK_010, *sseJ*: KK_011 and KK_012, *steA*: KK_013 and KK_014. The pSseF-2HA plasmid was constructed by amplifying the *sseABCDEFG* operon promoter the using primers KK_015 and KK_016. Expression and secretion of SseF is dependent on the chaperone protein SscB^23^. The coding regions of *sscB* and *sseF* were amplified using primers KK_017 and KK_018. The *sseABCDEFG* operon promoter was ligated to upstream end of *sscB* using Gibson Assembly. Each coding region plus native promoters was SalI/XhoI digested and ligated into SalI/XhoI digested pSopD2 with T4 DNA ligase (Invitrogen).

Plasmids pPipB2-2HA, pSteA-2HA, pSseJ-2HA, and pSseF-2HA were constructed from the pSopD2-2HA plasmid^21^. Briefly, pSopD2-2HA was SalI/XhoI digested. Protein coding regions plus 300–500 bp upstream of the coding region containing each gene’s native promoter were amplified from SL1344 genomic DNA, SalI/XhoI digested, and ligated into SalI/XhoI digested pSopD2-2HA with T4 DNA ligase (Invitrogen) resulting in a double hemagglutinin (HA)-tag at the C-terminal end of each effector all under the control of each effector’s native promoter. All cloned constructs were verified by DNA sequencing (Genewiz).

To generate the plasmid pACYC-2HA, the plasmid pPipB2-2HA was simultaneously amplified and linearized using primers KK_019 and KK_020. The entire plasmid except for amino acids 6–350 of PipB2 were amplified including: the entire backbone of pACYC184, the native promoter for *pipB2,* and the codons for the first 5 amino acids of PipB2. The amplified linearized plasmid was XhoI (NEB) digested and ligated back together, resulting in a re-circularized plasmid with truncated *pipB2* fused to the double HA tag under the control of the *pipB2* native promoter (pACYC-2HA). The resulting plasmid was transformed into *E. coli* DH10B for plasmid propagation and sequencing. Plasmid was sequenced (Genewiz) prior to transformation into the T3SS2^+^ *ssrB* overexpression strain (see below) resulting in the T3SS2^+^pACYC-2HA strain.

### Generation of T3SS2^+^ mutant

The chromosomal duplication of *ssrB* resulting in the T3SS2^+^ hypersecretion mutant was created by conjugating MFD*pir* transformed with the plasmid pRE112-*ssrB* with wild type SL1344 to make an unmarked chromosomal duplication of *ssrB* in the SL1344 chromosome between the genes *dppA* and SL1344_3597, specifically at the 3,837,749 base pair position according to the genome assembly NCBI_accession:NC_016810. Pro-conjugation single crossover mutants between pRE112-*ssrB* and the SL1344 chromosome were selected on LB agar plates containing chloramphenicol. Sucrose counter-selection was performed as described previously^24^ to select for the second crossover event, thus inserting *ssrB* into the chromosome and creating the T3SS2^+^ mutant. Unmarked complete deletion mutants in the T3SS2^+^ background were generated as described elsewhere^11^.

### Cell lines

HeLa cells (ATCC^®^ CCL-2™) were routinely cultured in normal growth media: Dulbecco’s Modified Essential Medium (DMEM) (Hyclone) containing 10% (v/v) heat-inactivated fetal bovine serum (FBS) (Gibco), 1% (v/v) Glutamax (Gibco), and 1% (v/v) nonessential amino acids (Gibco). Cells were maintained at 37 °C in a 5% CO_2_ atmosphere.

### HeLa cell infections for immunofluorescence

HeLa cells were infected as previously described^25^. In brief, HeLa cells were seeded on 12 mm diameter coverslips in 24-well plates at a density of 5 × 10^4^ cells/well, 16–24 h prior to infection. Overnight bacterial cultures were subcultured 1:33 in LB without antibiotics and incubated for 3 h at 37 °C with shaking. 1 mL of bacterial cultures was pelleted and resuspended in Dulbecco’s phosphate-buffered saline (DPBS) (Hyclone), diluted in DMEM, and added to the HeLa cells at a multiplicity of infection (MOI) of 100:1. The infection proceeded for 15 min at 37 °C in 5% CO_2_ before non-internalized bacteria were removed by three washes in DPBS and cells incubated in growth media containing 100 µg/mL gentamicin until 2 h post-infection, followed by growth media containing 10 µg/mL gentamicin for the remainder of the experiment. HeLa cells were infected for a total of 8 h.

### Antibodies for immunofluorescence

The primary goat polyclonal anti-*Salmonella* antibody CSA-1 (Kirkegaard and Perry Laboratories) was used at a dilution of 1:300. The mouse anti-LAMP1 antibody H4A3c developed by J.T. August and J.E.K. Hildreth, obtained from the Developmental Studies Hybridoma Bank developed under the auspices of the NICHD and maintained by the University of Iowa (Department of Biological Sciences, Iowa, USA), was used at a dilution of 1:300. The rabbit anti-Annexin A2 polyclonal antibody (PA5, ThermoFisher) was used at a dilution of 1:300. Alexa 647-labelled phalloidin and secondary antibodies were obtained from Thermo Fisher Scientific and used at a dilution of 1:500: Alexa plus 405-conjugated donkey anti-goat, Alexa 488-conjugated donkey anti-mouse, Alexa 568-conjugated donkey anti-goat, and Alexa 568-conjugated donkey anti-rabbit.

### Immunofluorescence microscopy

Cells were fixed, permeabilized, and stained essentially as previously described^11,26^. In brief, cell monolayers seeded on glass coverslips were fixed with 4% (v/v) paraformaldehyde in DPBS at room temperature for 10 min and washed three times in DPBS. Excess paraformaldehyde was quenched with 50 mM ammonium chloride for 10 min at room temperature followed by two washes in DPBS. Cells were permeabilized in ice-cold acetone for 5 min at − 20 °C and then blocked in 1% bovine serum albumin (BSA, wt/vol) (Sigma) in DPBS for 30 min at room temperature. Cells on coverslips were then incubated with primary antibodies diluted in 1% BSA in DPBS at room temperature for 1 h followed by three washes in DPBS. Secondary antibodies diluted in 1% BSA in DPBS were added to the coverslips and incubated at room temperature for 1 h and then washed once with DPBS. Cells were then incubated for 10 min at room temperature with DAPI (Invitrogen) diluted in DPBS followed by two DPBS washes. Cells were then washed in deionized water prior to mounting with ProLong Gold Antifade Mountant (Life Technologies) on glass slides. Microscopy was performed using Olympus IX81 (100× objective) and Zeiss Axio Imager M2 (100× objective) microscopes using SlideBook 4.1.0 and Zeiss Zen Pro softwares, respectively. Images were further analyzed with ImageJ version 2.1.0/1.53c and Adobe Photoshop version 21.0.1. Confocal microscopy was performed using a Leica SP8 confocal microscope (100× objective) using Leica Application Suite X software and Adobe Photoshop version 21.0.1.

To quantify the number of infected cells with LAMP1^+^-tubules, we imaged at least one infected cell per field of view, then quantified the number of infected and uninfected cells per field of view. Infected cells were then scored for the presence or absence of LAMP1^+^-tubules radiating outwards from labelled *Salmonella*. At least 60 infected cells per strain were scored blind in each experiment, and each experiment was repeated at least three times.

To quantify the distance between *Salmonella* and the nuclei of infected cells, immunofluorescent images were analyzed by a custom CellProfiler software (version 4.2.1)^27^ pipeline. Host nuclei (stained blue) and *Salmonella* (red) were identified as primary objects and the minimum distance (edge to edge) between the two was measured (in µM) and enumerated. At least 200 *Salmonella*-to-nucleus distances were measured, with three replicates.

### Annexin A2 knockdown in HeLa cells and *Salmonella* infection

Human AnxA2 expression was silenced via RNA interference (RNAi) using commercially available AnxA2 small hairpin RNA (shRNA) lentiviral particles (sc-270151-V, Santa Cruz Biotechnology). Scrambled control shRNA lentivirus particles were used as a negative control (sc-108080, Santa Cruz Biotechnology). Briefly, HeLa cells in twelve-well tissue culture plates at approximately 50% confluency were treated with DMEM containing 10 µg/mL of Polybrene (sc-134220, Santa Cruz Biotechnology) and infected by adding shRNA lentiviral particles at a MOI of 1 for 24 h, following the manufacturer’s instructions. To select stable cells expressing the shRNA, HeLa cells were treated with puromycin dihydrochloride (sc-108071, Santa Cruz Biotechnology) at a final concentration of 10 µg/mL. Knockdown efficiency was assessed by western blotting of whole-cell lysates using rabbit α-Annexin A2 polyclonal antibodies (PA5, ThermoFisher), with Beta Tubulin used as a loading control and probed with mouse α-Beta Tubulin antibodies (Sigma). These AnxA2 knockdown and control cells were then infected with *S.* Typhimurium, processed for immunofluorescence microscopy, and stained for *Salmonella*, LAMP1, and DNA/nucleus as described above, as well as actin with Alexa 647-labelled phalloidin.

### SILAC labelling

HeLa cells were split from normal growth media into arginine and lysine-free DMEM (Caisson Laboratories Inc.) supplemented with 10% heat inactivated dialyzed FBS (Gibco) and either 1 mM ^2^H_4_-lysine and 0.1 mM ^13^C_6_-arginine (Cambridge Isotope Laboratories) for heavy cells or normal isotopic abundance of l-lysine and l-arginine (Sigma) for light cells. Cells were maintained in labelling media for at least 5 cell divisions to ensure complete labelling as described elsewhere^28^.

### *Salmonella* infection of SILAC-labelled HeLa cells and analyses

Light and heavy labelled HeLa cells were seeded at a density of approximately 4.5 × 10^6^ cells per 15 cm culture plate 16–24 h prior to infection. For all HeLa cell infections: overnight bacterial cultures were subcultured 1:33 in LB without antibiotics and incubated for 3 h at 37 °C with shaking. 1 mL of bacterial cultures was pelleted and resuspended in Dulbecco’s Phosphate-Buffered Saline (DPBS) (Hyclone), diluted in the appropriate isotope-supplemented DMEM, and added to the HeLa cells at a multiplicity of infection (MOI) of ≈ 100:1. Infections proceeded for 15 min at 37 °C in 5% CO_2_, after which non-internalized bacteria were removed by three washes in DPBS and cells incubated in growth media containing 100 µg/mL gentamicin until 2 h post-infection, followed by growth media containing 10 µg/mL gentamicin for the remainder of the experiment. HeLa cells were infected for a total of 8 h.

See Supplemental Fig. S1 for a diagram of the experimental design. For two of the three replicate experiments, seven 15 cm cell culture plates of light labelled HeLa cells were infected with one of T3SS2^+^*∆sifA*-pSifA-2HA, T3SS2^+^*∆sopD2*-pSopD2-2HA, T3SS2^+^*∆pipB2*-pPipB2-2HA, T3SS2^+^*∆steA*-pSteA-2HA, T3SS2^+^*∆sseJ*-pSseJ-2HA, T3SS2^+^*∆sseF*-pSseF-2HA, or T3SS2^+^pACYC-2HA (vector control) (Table 1), while seven 15 cm cell culture plates of heavy labelled HeLa cells were infected with T3SS2^+^-pACYC-2HA. For the third experimental replicate, a label swap was performed wherein seven 15 cm cell culture plates of light labelled HeLa cells were infected with T3SS2^+^pACYC-2HA and seven 15 cm cell culture plates of heavy labelled HeLa cells were infected with one of T3SS2^+^*∆sifA*-pSifA-2HA, T3SS2^+^*∆sopD2*-pSopD2-2HA, T3SS2^+^*∆pipB2*-pPipB2-2HA, T3SS2^+^*∆steA*-pSteA-2HA, T3SS2^+^*∆sseJ*-pSseJ-2HA, T3SS2^+^*∆sseF*-pSseF-2HA, or T3SS2^+^-pACYC-2HA. This label swap allowed us to control for interference by the heavy or light isotopes used for SILAC labelling.

Infected light and heavy labelled cells were washed three times in ice cold DPBS and manually detached from the culture dish containing 5 mL ice-cold DPBS and transferred into 15 mL tubes. Cells were spun at 300×*g* for 10 min in a centrifuge set to 4 °C. The supernatant was removed, and cell pellets were resuspended in RIPA buffer (25 mM Tris–HCl pH 7.6, 150 mM NaCl, 1% NP-40, 1% sodium deoxycholate, 0.1% SDS, supplemented with mini cOmplete protease inhibitor cocktail (Roche)) and incubated on ice for 10 min. Cell lysates were centrifuged for 20 min in a 4 °C microcentrifuge at maximal speed to pellet cell debris. Protein concentrations were determined using a Bicinchoninic Acid Assay (BCA) (Sigma). Lysates from cells infected with each of the seven different T3SS2^+^ strains were then mixed at a 1:1 ratio with lysate from the opposite SILAC labelled HeLa cells infected with the vector control T3SS2^+^-pACYC-2HA. e.g., lysate from light labelled HeLa cells infected with T3SS2^+^*∆sifA*-pSifA-2HA was combined at a 1:1 ratio with lysate from heavy labelled HeLa cells infected with T3SS2^+^-pACYC-2HA. 25 µL of anti-HA magnetic beads (Thermo Fisher Scientific) was added to the mixed lysates and incubated with end-over-end rotation overnight at 4 °C. Immunoprecipitated proteins were eluted from the magnetic beads using a basic elution protocol according to manufacturer’s specifications. Eluted proteins were separated by SDS-PAGE, bands excised, and subjected to in-gel tryptic digestion and proteomic analyses as previously described^29^.

### Mass spectrometry data analyses

The resulting peptides of the immunoprecipitated proteins after in-gel tryptic digestion were analyzed by liquid chromatography-tandem mass spectrometry (LC–MS/MS) using a Bruker Impact II Qtof as described elsewhere^27^. Peptides were searched on MaxQuant version 1.6.7.0^30^ against UniProt sequence database (uniport.org) for *Homo sapiens* (taxonomy 9606: downloaded on April 12, 2019 with 169,993 entries), *S.* Typhimurium (downloaded on November 15, 2015 with 38,842 entries), and software provided common contaminants (246 entries). The mass spectrometry proteomics data have been deposited to the ProteomeXchange Consortium via the PRIDE^28^ partner repository with the dataset identifier PXD025582.

### In vitro secretion assays

#### Protein secretion assays

Effectors secreted by SPI-1 encoded type III secretion system (T3SS1) were analyzed by way of a SPI-1 secretion assay as described elsewhere^31,32^. Overnight cultures of *S.* Typhimurium strains in LB-broth were subcultured 1:100 in fresh LB broth containing no antibiotics. Cultures were grown at 37 °C for 6 h with shaking after which the optical density at 600 nm was measured. 3 mL of culture were centrifuged at 9500×*g* for 10 min at 4 °C. Culture supernatant was passed through a 0.22 µm filter, precipitated with trichloroacetic acid (TCA; Sigma) at a final concentration of 10% (v/v), and incubated on ice at 4 °C overnight.

Effectors secreted by T3SS2 were analyzed by way of a SPI-2 secretion assay as previously described^33^. 1 mL of overnight *S.* Typhimurium cultures was washed twice in a low phosphate and low magnesium-containing medium (LPM), then inoculated at a 1:50 dilution in 30 mL of LPM at a pH of 5.8. LPM composition was 5 mM KCl, 7.5 mM (NH_4_)_2_SO_4_, 0.5 mM K_2_SO_4_, 80 mM MES, 38 mM glycerol (0.3% v/v), 0.1% casamino acids, 24 µM MgCl_2,_ 337 µM PO_4_^3−^. Cultures were grown at 37 °C with shaking for 9 h after which the optical density at 600 nm was measured. Bacteria were collected by centrifugation for 30 min at 3000×*g* in a 4 °C centrifuge. Culture supernatant was passed through a 0.22 µm filter, precipitated with TCA at a final concentration of 10% (v/v), and incubated on ice at 4 °C overnight.

#### Analysis of secreted proteins

For T3SS1-secreted proteins: The TCA insoluble fraction was collected by centrifugation for 45 min at 13,000×*g* at 4 °C. Precipitate pellets were then washed in ice cold acetone and centrifuged at maximal speed in a 4 °C microfuge. Pellets were air-dried before resuspension in a volume of 2× SDS-PAGE sample buffer (100 mM Tris–HCl, pH 6.8, 20% glycerol, 4% SDS, 0.002% bromophenol blue, and 200 mM dithiothreitol) normalized to A_600_ of the original culture and boiled for 10 min. Samples were separated by SDS-PAGE using 12% polyacrylamide gels and stained with Coomassie Brilliant Blue R-250.

For T3SS2-secreted proteins: The TCA insoluble fraction was collected by centrifugation for 45 min at 14,000×*g* at 4 °C. Pellets were solubilized with a volume of 2× SDS-PAGE sample buffer adjusted according to the A_600_ of the original culture.

Proteins from equivalent numbers of bacterial cells, as determined by the A_600_, were separated on SDS-12% polyacrylamide gels, transferred to Pure Nitrocellulose membranes (Bio-Rad) using a wet transfer cell. Membranes were blocked in blocking buffer (Tris-buffered saline containing 0.1% (v/v) Tween 20 (TBS-T) and 5% (w/v) non-fat milk) overnight at 4 °C. Blots were incubated with the following primary antibodies in blocking buffer for one hour at room temperature: rabbit affinity-purified antibodies raised against recombinant SseB and SseD (1:1500), rat anti-HA monoclonal antibody (1:2000, Roche), or mouse anti-beta-tubulin (1:1500, Abcam). Three 5-min wash steps were performed using TBS-T followed by room temperature incubation of membranes with secondary antibodies. Secondary antibodies conjugated to horseradish peroxidase (HRP; goat anti-rat, goat anti-rabbit, or goat anti-mouse) were diluted to 1:5000 in blocking buffer. Antibody complexes were detected using ClairtyTM Western ECL Substrate (Bio-Rad Laboratories, Inc) prior to detection in a BioRad Gel Imaging System.

### Pull-down assays

Strains T3SS2^+^*∆sopD2*-pSopD2-2HA, T3SS2^+^*∆pipB2*-pPipB2-2HA, and T3SS2^+^-pACYC-2HA were grown in LB for 9 h at 37 °C with shaking. SPI-2 expression was induced by washing cell pellets from cultures twice in LPM (low phosphate and low magnesium-containing medium; 5 mM KCl, 7.5 mM (NH_4_)_2_SO_4_, 0.5 mM K_2_SO_4_, 80 mM MES, 38 mM glycerol (0.3% v/v), 0.1% casamino acids, 24 µM MgCl_2,_ 337 µM K_2_HPO_4_/KH_2_PO_4_ (pH 7.4)^33^) prior to inoculation at a 1:50 dilution in 400 mL of LPM at a pH of 5.8. Cells were pelleted by centrifugation and culture supernatant was filter sterilized by passage through a 0.22 µm filter. Sterilized culture supernatant was concentrated by passage through a 10 kDa molecular weight cut-off centrifugal filter unit (Millipore Sigma). Protein concentration of the concentrated culture supernatant was determined by BCA assay. Approximately 20 µg of concentrated culture supernatant was mixed with 5 µg of recombinant human His_6_-tagged annexin A2 (Novus Biologicals) and incubated with end-over-end rotation at 4 °C overnight. Samples were incubated with Ni-Sepharose High Performance Beads (Sigma) for two hours at room temperature. Beads were washed four times with TBS-T (20 mM Tris–HCL, 150 mM NaCl, 0.75% Triton X-100), resuspended in 160 µL SDS-PAGE loading buffer, and boiled for 10 min prior to Western blot analysis.

### Western blotting

Samples were separated on 12% polyacrylamide gels and transferred to Pure Nitrocellulose (Bio-Rad) using a wet transfer cell. Membranes were blocked with blocking buffer (5% w/v skim milk powder in Tris-buffered saline containing 0.1% Tween-20 (TBS-T)) overnight at 4 °C. Membranes were incubated with the following primary antibodies diluted in blocking buffer for 1 h at room temperature: α-HA (rat; Roche) diluted 1:2000, mouse α-beta tubulin (Abcam) diluted at 1:1500, and α-Annexin A2 Clone 5 (mouse; BD Biosciences) diluted 1:2000. Membranes were washed three times for five minutes each with TBS-T prior to incubation with the following secondary antibodies diluted in blocking buffer for one hour at room temperature: HRP-conjugated goat α-rat or goat α-mouse (Sigma) diluted 1:5000. Membranes were washed as described above and treated with ClarityTM Western ECL Substrate (Bio-Rad) prior to chemiluminescent developing.

### Data analysis

Mass spectrometry data were analyzed in Perseus version 1.6.7.0. STRING analysis was performed using the STRING database online tool version 11.0 (https://string-db.org/). Graphs were made using Prism9 (GraphPad).

### Statistical analyses

Statistical analysis was performed using Prism9 (GraphPad). Analysis was performed using a Mann–Whitney test to compare two groups and two-way ANOVA for more than one group with Tukey’s multiple comparison test as appropriate. Aggregate results represent the mean ± standard error on the mean.

## Results

### Overexpression of the SsrB regulator enables identification of SPI-2 effectors during infection

A barrier to identifying specific and significant T3SS2-secreted effector interactions is that the quantity of T3SS2-secreted effectors pales in comparison to the abundance of both T3SS1-secreted effectors and host proteins. This hampers identification of T3SS2-effector binding partners as their low abundance confounds separation of specific binding partners from the background noise of mass spectrometry. It is therefore inadequate to rely on native expression of T3SS2 effectors to identify binding partners. To mitigate this limitation, we created an isogenic strain of *S.* Typhimurium SL1344 that specifically secretes increased amounts of T3SS2-secreted effectors, but a similar amount T3SS1-secreted effectors relative to wild type SL1344.

The two-component regulatory system SsrA/SsrB encoded within SPI-2 is a major regulator of virulence associated events linked to SPI-2. The response regulator SsrB binds to all promoters within SPI-2 and is indispensable for expression of the T3SS2 secretion apparatus and its associated effectors encoded within SPI-2, as well as effectors encoded outside SPI-2^34–37^. Overexpression of plasmid-encoded SsrB has previously led to discovery of additional T3SS2-secreted effectors within an in vitro T3SS2-secretome analysis^38^ but *ssrB* overexpression has to our knowledge never been used to identify effector-host binding partners. *ssrB* was overexpressed by duplicating *ssrB* including its native promoter within the chromosome of SL1344 in an intergenic region distant from SPI-2 and between the genes *dppA* and SL1344_3597 (a putative xanthine permease). The *ssrB* overexpression strain will hereafter be referred to as the T3SS2^+^ strain.

**Figure 1:**
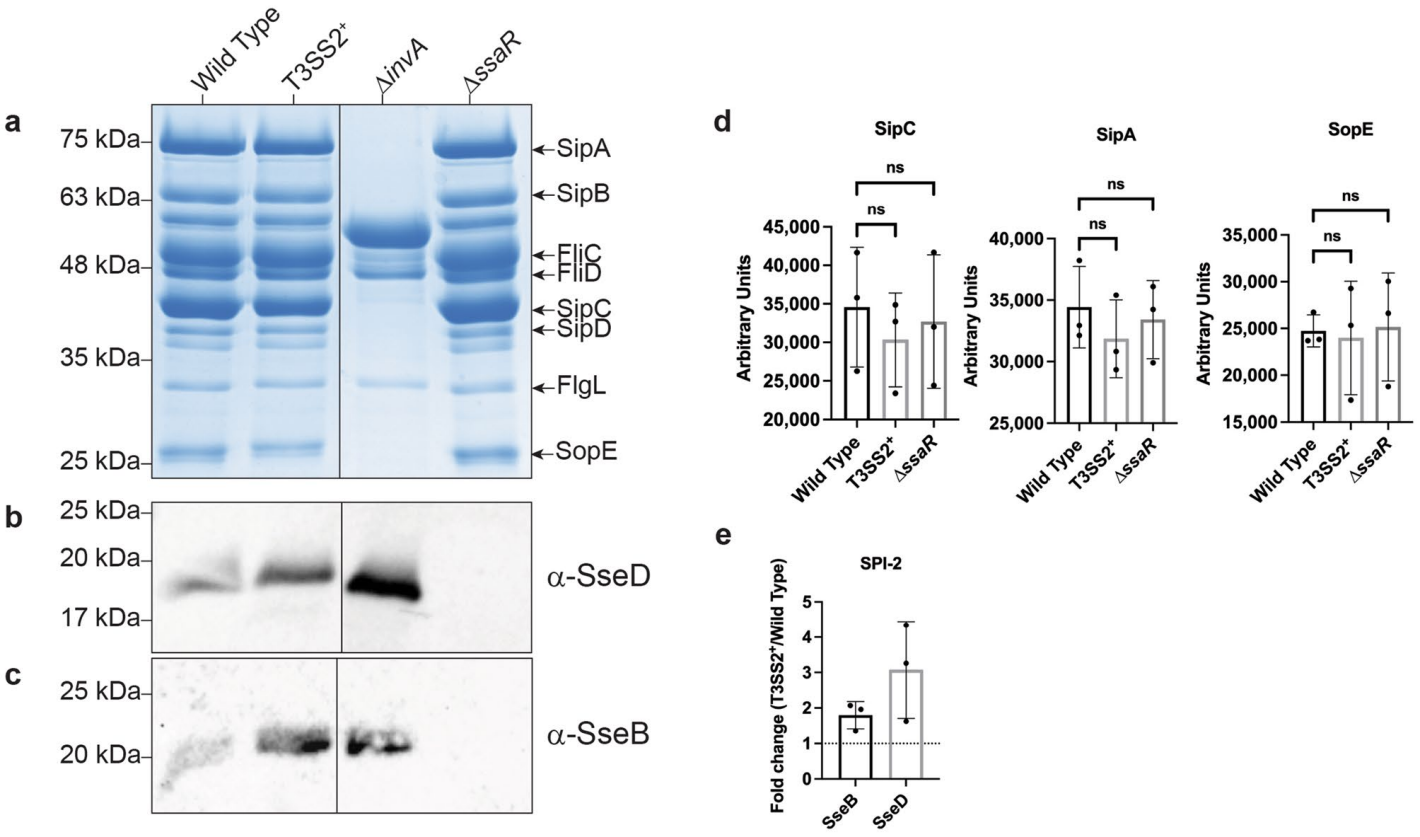
*ssrB* overexpression increases T3SS2 secretion, but not T3SS1 secretion. Images shown are representative of *n* = 3 experiments. **(a)** Secreted proteins from indicated strains grown under SPI-1 inducing conditions were separated by SDS-PAGE and stained with Coomassie Brilliant R250. **(b,c)** Secreted proteins from indicated strains grown under SPI-2 inducing conditions were precipitated from sterilized culture supernatant and separated on an SDS-PAGE gel and immunoblotted with α-SseD **(b)** or α-SseB **(c)**. **(a–c)** Images were cropped where indicated by dividing lines. Original Coomassie stained SDS-PAGE gel and western blots are shown in Supplemental Fig. S2. **(d)** Densitometric analysis of SPI-1 secreted effectors SipA, SipC, and SopE as determined by 3 SDS-PAGE gels stained by Coomassie. *Ns* not significant as determined by a Kruskal–Wallis test with Dunn’s correction for multiple comparisons. **(e)** Fold change determined by densitometry of 3 Western blots from independent experiments. Mean fold change ± standard deviation is shown. Fold change = (Densitometry of T3SS2^+^)/(Densitometry of wild type). Dashed line = 1.

Effector secretion by the T3SS1 and T3SS2 was assessed by SPI-1 and SPI-2 secretion assays, respectively. SPI-1 inducing growth conditions that induce T3SS1-secretion also induce the secretion of flagellar proteins FliC, FliD, and FlgL (Fig. 1a). Overexpression of *ssrB* in the T3SS2^+^ strain neither increased nor decreased T3SS1-secretion relative to wild type (Fig. 1a,d). The *ΔinvA* is a negative control strain^39^ as it has a non-functional T3SS1-secretion apparatus, but still secretes flagellin proteins into the culture supernatant in a T3SS1-independent manner. The T3SS2-apparatus mutant *ΔssaR*^19^ secretes approximately equivalent amounts of T3SS1-effectors as both wild type and T3SS2^+^ strains (Fig. 1a,d).

The SPI-2 encoded and secreted proteins SseB and SseD (filament and pore forming components of the T3SS2 secretion apparatus, respectively) provide a convenient means to assess T3SS2 secretion^33^. Growth of bacteria under SPI-2 inducing conditions indicates that the T3SS2^+^ strain secretes increased amounts of both SseD (Fig. 1b) and SseB (Fig. 1c) relative to wild type (Fig. 1e). The *ΔssaR* mutant failed to secrete either SseB or SseD. In all, *ssrB* overexpression did not impact T3SS1-secretion but did increase T3SS2-secretion.

**Figure 2:**
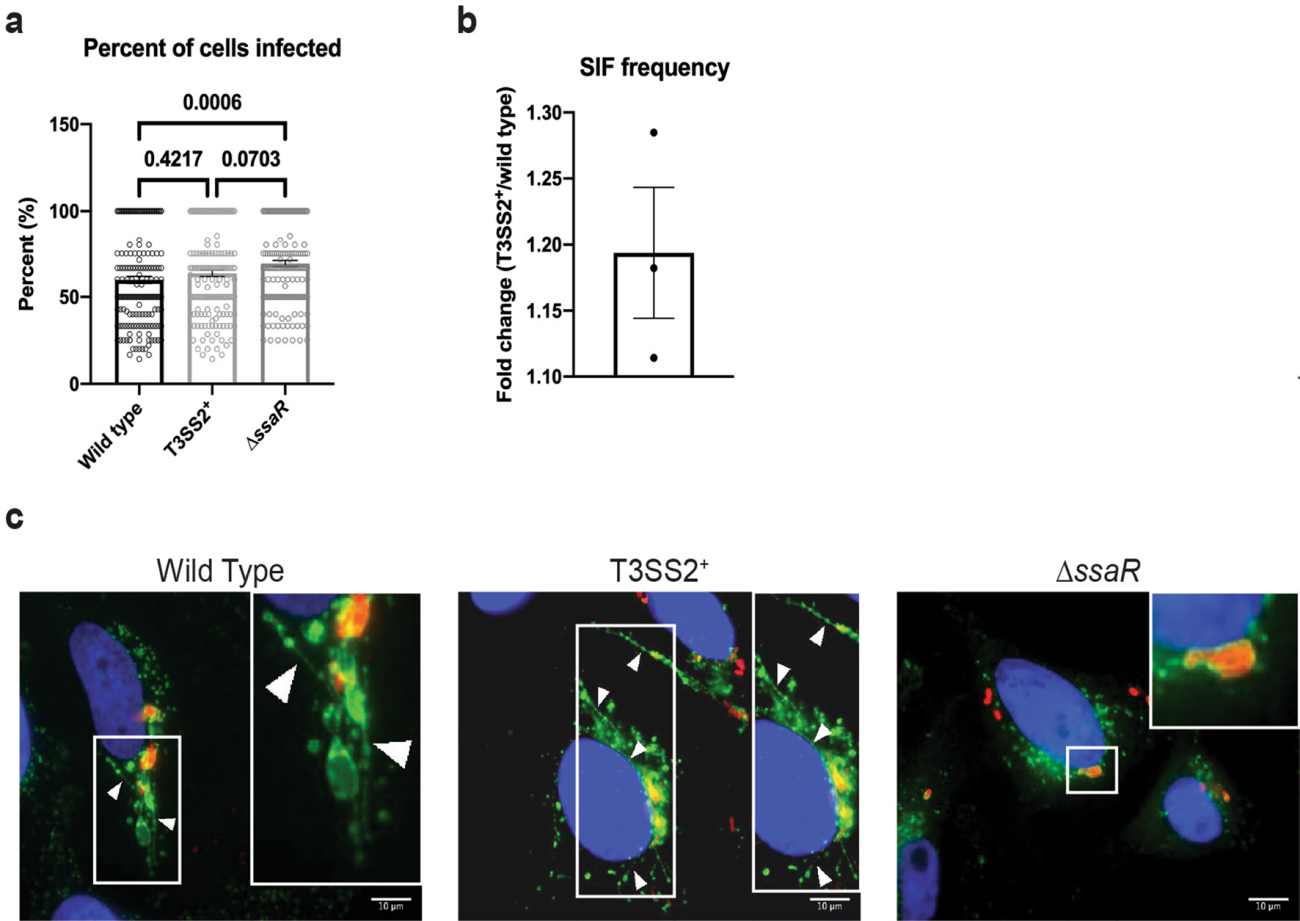
T3SS2^+^ infected HeLa cells exhibit increased SIF frequency. HeLa cells were infected at an MOI ≈ 100 with the indicated strains for 8 hours prior to cell fixation. Cells were immunostained for *Salmonella* (red) and LAMP1 (green), and the nucleus was stained with DAPI (blue). 60 distinct fields of view were used for quantification with at least 1 infected cell per field of view for each experiment. **(a)** Percentage of cells infected as determined by enumerating the number of both infected and uninfected cells in each field of view. Mean ± standard error of the mean is shown (*n* = 3). Significance determined by a Kruskall–Wallis test with Dunn’s multiple comparisons post-test. *p-*values are as indicated on graph. **(b)** Percent of infected cells with SIFs determined by enumerating the number of infected cells and the number of infected cells with SIFs per field of view. Mean ± standard error of the mean is shown (*n* = 3). **(c)** Representative images of HeLa cells infected with a MOI ≈ 100 with indicated strain at 8 h.p.i. White boxes indicate zoomed-in region in inset. Arrowheads indicate LAMP1^+^-tubules. Scale Bar = 10 µm.

As *ssrB* overexpression does not appear to have an effect on T3SS1 secretion we hypothesized that increased T3SS2-secretion in the T3SS2^+^ strain would affect primarily T3SS2-, but not T3SS1-, mediated processes. It follows then that increased T3SS2-secretion should increase T3SS2-mediated events such as increased SIF biogenesis frequency during infection. The T3SS2^+^ strain does not infect a significantly higher proportion of HeLa cells relative to wild type and the T3SS2-defective *ΔssaR* strain (Fig. 2a,c). T3SS2^+^-infected cells have a significantly increased incidence of SIF biogenesis (30.40 ± 2.54% of infected cells with SIFs) relative to wild type infected cells (24.94 ± 2.61% of infected cells with SIFs) (Fig. 2b,c). Thus, *ssrB* overexpression does not appear to affect T3SS1-secretion or T3SS1-mediated processes such as invasion, but does increase T3SS2-secretion (Fig. 1) and the incidence of SIF biogenesis (Fig. 2), a T3SS2-mediated event.

**Figure 3:**
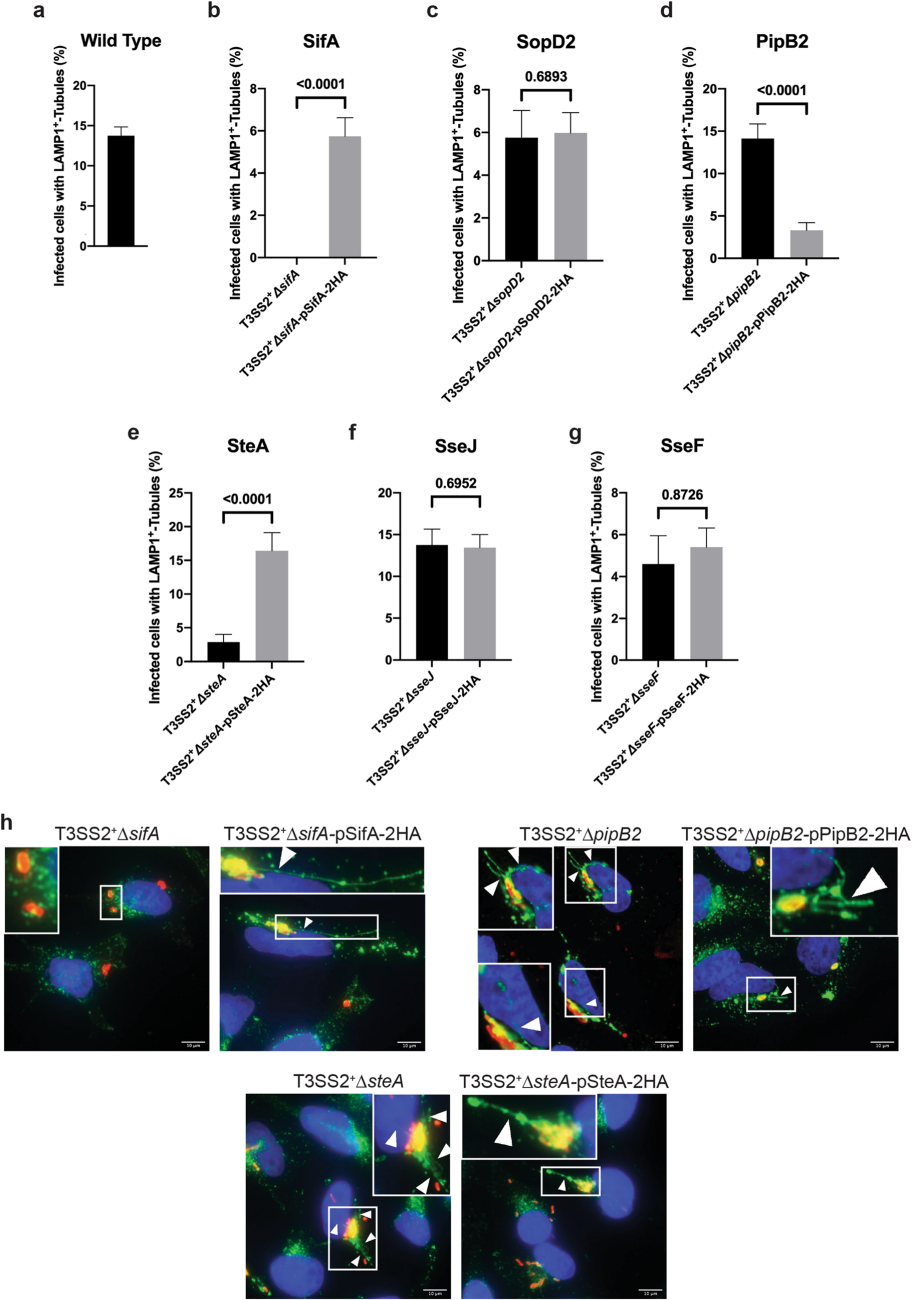
Complementation of SIF biogenesis. HeLa cells were infected at an MOI ≈ 100 with the indicated strains for 8 hours prior to cell fixation. Cells were immunostained for *Salmonela* (red) and LAMP1 (green), and the nucleus was stained with DAPI (blue). 60 distinct fields of view were used for quantification with at least 1 infected cell per field of view for each experiment. **(a–g)** Quantification of LAMP1^+^-tubule frequency (SIFs) in HeLa cells infected with single effector deletion mutants in the T3SS2^+^ background and their corresponding complemented strains after 8 h of infection. The average frequency of infected cells with LAMP1^+^-tubules ± standard error of the mean is shown (*n* = 3). Strains were analyzed in a single repeated experiment but have been divided into separate panels for the sake of clarity. At least 60 infected cells per strain were blindly analyzed in each experiment. *p*-values are as indicated as determined by two-tailed Mann–Whitney test with a 95% confidence level. **(h)** Representative images for select strains. White boxes indicate zoomed-in region in inset. Arrowheads indicate LAMP1^+^-tubules. Scale Bar = 10 µm.

Single effector deletions of our six effectors of interest (SifA, SopD2, SteA, PipB2, SseJ, and SseF) were generated in the T3SS2^+^ mutant background. These six effectors were chosen for their purported—yet ill-defined—roles in SIF biogenesis and SCV membrane maintenance. Each effector gene, except that for SifA which has an internal tandem HA tag (Table 2), was cloned into a C-terminal HA-tagging vector under the control of their respective native promoters. The resulting constructs were then assessed for complementation of the SIF phenotype defect of the corresponding *S.* Typhimurium single effector knockout strains in infected HeLa cells (Fig. 3). SIF phenotype complementation has previously been used to validate the functionality of T3SS2-secreted effector fusion proteins^40^. Complementation of T3SS2^+^Δ*sifA* and T3SS2^+^Δ*steA* with pSifA-2HA and pSteA-2HA, respectively, significantly increased the frequency of SIF biogenesis in infected cells relative to their uncomplemented counterpart strains (Fig. 3b,e, respectively).

There was no significant difference in the frequency of SIF biogenesis in infected cells with complemented T3SS2^+^Δ*sopD2*, T3SS2^+^Δ*sseJ*, and T3SS2^+^Δ*sseF* compared to their respective uncomplemented counterpart strains (Fig. 3c,f,g, respectively). Deletion of *sseJ* does not significantly alter SIF frequency relative to wild type infected cells^11,41,42^; therefore, complementation with T3SS2^+^Δ*sseJ* with pSseJ-2HA should have negligible effect on the percentage of infected cells with SIFs–consistent with our findings (Fig. 3f). There was a small insignificant increase in the proportion of infected cells with SIFs in the complemented T3SS2^+^Δ*sseF* strain versus the uncomplemented counterpart strain (Fig. 3g). We and others have previously demonstrated that *sseF* deletion dramatically reduces SIF frequency relative to wild type^11,43,44^, so complementation of T3SS2^+^Δ*sseF* should increase SIF frequency relative to the uncomplemented strain. SseF-2HA is readily expressed during infection of HeLa cells with T3SS2^+^Δ*sseF* (Fig. S3a,b), thus the insignificant evidence of complementation of T3SS2^+^Δ*sseF* is not due an expression deficiency, but may be due to a translocation failure during infection. It is also possible that proper function of SseF requires coordinated expression of SseG^44–46^, and ectopic overexpression of SseF on a plasmid may disrupt this cooperation.

There was no increase in the frequency of infected cells with SIFs in the complemented T3SS2^+^Δ*sopD2* strain versus its uncomplemented counterpart strain (Fig. 3c) and a decrease in the frequency of infected cells with SIFs in the complemented T3SS2^+^Δ*pipB2* strain versus its uncomplemented counterpart (Fig. 3d), despite the fact that both SopD2-2HA and PipB2-2HA are secreted in vitro (Supplemental Fig. S3a,b) and translocated into HeLa cells during infection (Supplemental Fig. S3e,f). Other groups have successfully complemented the SIF phenotype in a Δ*sopD2* mutant using the same pSopD2-2HA plasmid^7,21^, so it is unknown why pSopD2-2HA did not complement SIF frequency in the T3SS2^+^Δ*sopD2* strain in our hands. One possibility is that simultaneous overexpression of SsrB and SopD2-2HA may change the secretion hierarchy and stoichiometry of T3SS2 effectors and alter the dynamics of SIF biogenesis. Consistent with a previous report, strains complemented with a plasmid-borne copy of PipB2-2HA reduces SIF frequency in infected cells likely owing to overexpression of PipB2^5^. Expression of *pipB2* at wild type levels promotes SIF extension away from the SCV, but *pipB2* overexpression induces accumulation of late endosomal and lysosomal compartments at the cell periphery, thereby reducing the availability of membrane components required for SIF biogenesis and reducing the frequency of SIF biogenesis^5^. In conclusion, most HA-tagged effector constructs successfully complemented the SIF phenotype defect observed in their respective uncomplemented counterpart strains.

### SopD2 and PipB2 share common interaction partners

We set out to systematically investigate effector-effector and effector-host protein interactions during *S.* Typhimurium infection through quantitative proteomic analysis of *S*. Typhimurium infected SILAC-labelled HeLa human epithelial cells. Briefly, HeLa cells were infected with the T3SS2^+^ single deletion mutants complemented with plasmid encoded HA-tagged effectors (see Supplemental Fig. S1 for experimental design). Pull-down of HA-tagged effectors and interacting proteins was performed by immunoprecipitation against the each tagged effector’s HA tag (effector-IP). Immunoprecipitated complexes were then profiled by mass spectrometry. In total, we identified 554 different proteins originating from either the human or *S.* Typhimurium proteome after removal of common contaminants. Proteins were further gated for those with infection/control SILAC ratios of at least 0.66. Proteins with SILAC ratios of at least 0.66 were likely pulled-down (i.e., relatively abundant as compared to the tag-only control) and not part of the background noise associated with mass spectrometry. Thus, these proteins demonstrate a relative difference in quantity between control and infection, thus indicating enrichment of that protein and suggestive of an interaction with the tagged effector. 273 proteins passed this threshold which were then used for subsequent analysis.

**Figure 4:**
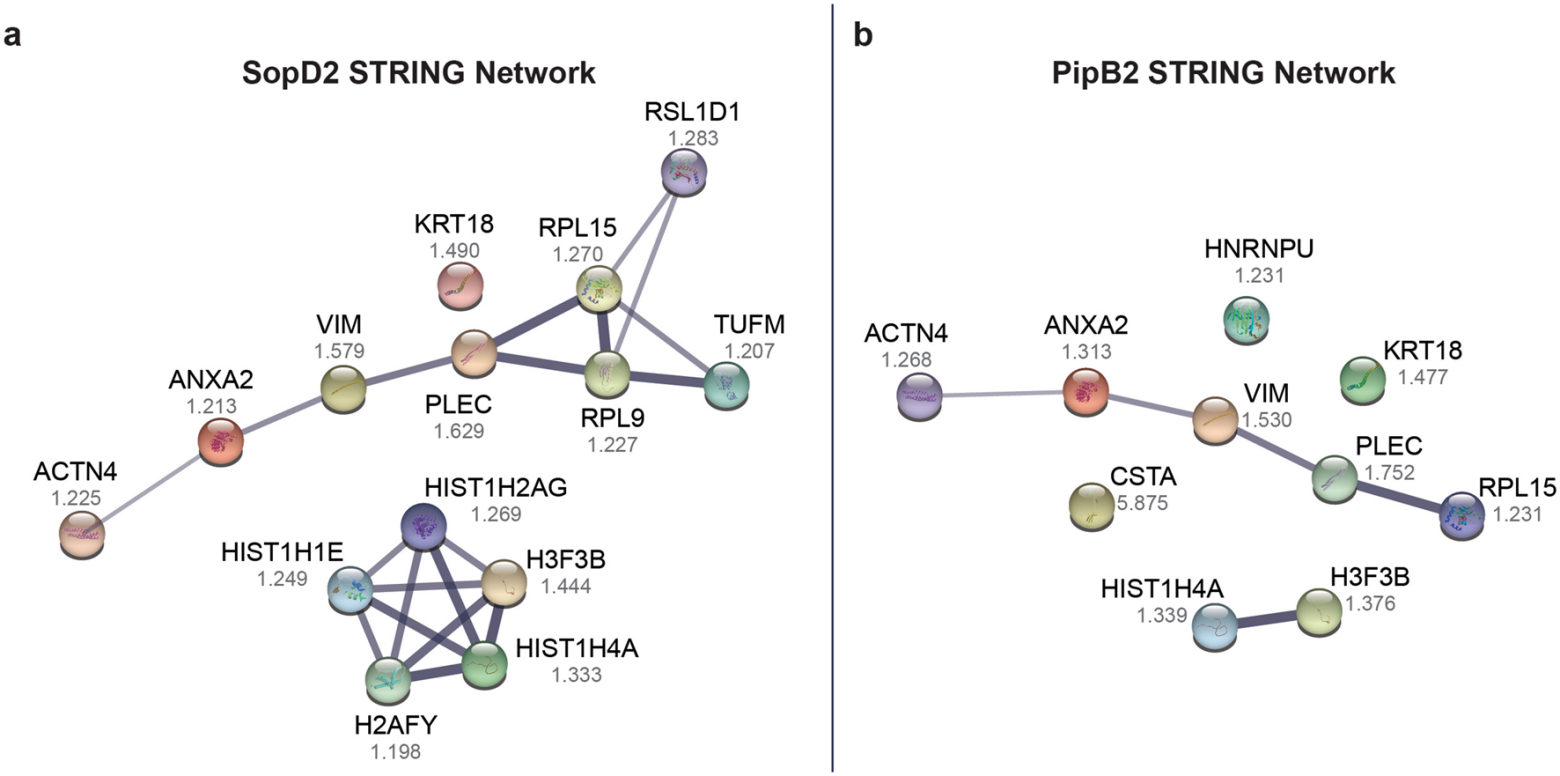
SopD2 and PipB2 immunoprecipitation reveals common proteins that are directly or indirectly linked. STRING analysis on the top hits from the SopD2-IP **(a)** and PipB2-IP **(b)** as identified by mass spectrometry. Median SILAC ratios are shown for each protein as determined from 3 independent experiments. STRING networks show both functional and physical protein associations. Line thickness indicates the strength of data supporting the interaction with a minimum confidence of interaction score of 0.4.

We then gated for proteins with SILAC ratios approximately greater than 1.2 to differentiate from non-specific protein interactions (see Supplementary Data for full list of proteins). Proteins with SILAC ratios greater than 1.2 is indicative of having a greater abundance or stronger interaction relative the control samples. Furthermore, proteins with SILAC ratios greater than 1.2 include the top 10% of largest SILAC ratios for these experiments. Next, we performed STRING analysis^47^ on these proteins for each of the six effector-IPs to identify trends or common interaction partners or networks amongst the effector-IPs. STRING is a database of known protein–protein interactions including both direct and indirect associations. Direct associations are protein–protein binding events and indirect associations are proteins interacting together on a similar pathway, but not directly binding to each other. STRING analysis of the proteins with SILAC ratios greater than 1.2 from the SopD2-IPs and PipB2-IPs revealed several identified proteins in common that may interact either directly or indirectly (Fig. 4). Specifically, the proteins alpha-actinin-4, annexin A2 (AnxA2), vimentin, and plectin appear to form a series of direct or indirect protein–protein interactions. Each of these four proteins were consistently enriched in both the SopD2- and PipB2-IPs, suggesting that a multi-protein complex between SopD2, PipB2, and these host proteins may form.

Vimentin was found with a median 1.579-fold and 1.530-fold increase in SopD2 and PipB2 IPs relative to control, respectively. AnxA2 was found with a median 1.213-fold and 1.313-fold increase in SopD2 and PipB2 IPs relative to control, respectively. Alpha-actinin-4 was found with a median 1.225-fold and 1.268-fold increase in SopD2 and PipB2 IPs relative to control, respectively. Finally, plectin was found with a median 1.629-fold and 1.752-fold increase in SopD2 and PipB2 IPs relative to control, respectively. It is important to note that vimentin and alpha-actinin-4 were also found with SILAC ratios around 1 in Control_Heavy_:Control_Light_ samples (see Supplementary Data) suggesting that vimentin and alpha-actinin-4 may non-specifically bind to the anti-HA coated magnetic beads.

### SopD2 binds annexin A2 in vitro

**Figure 5:**
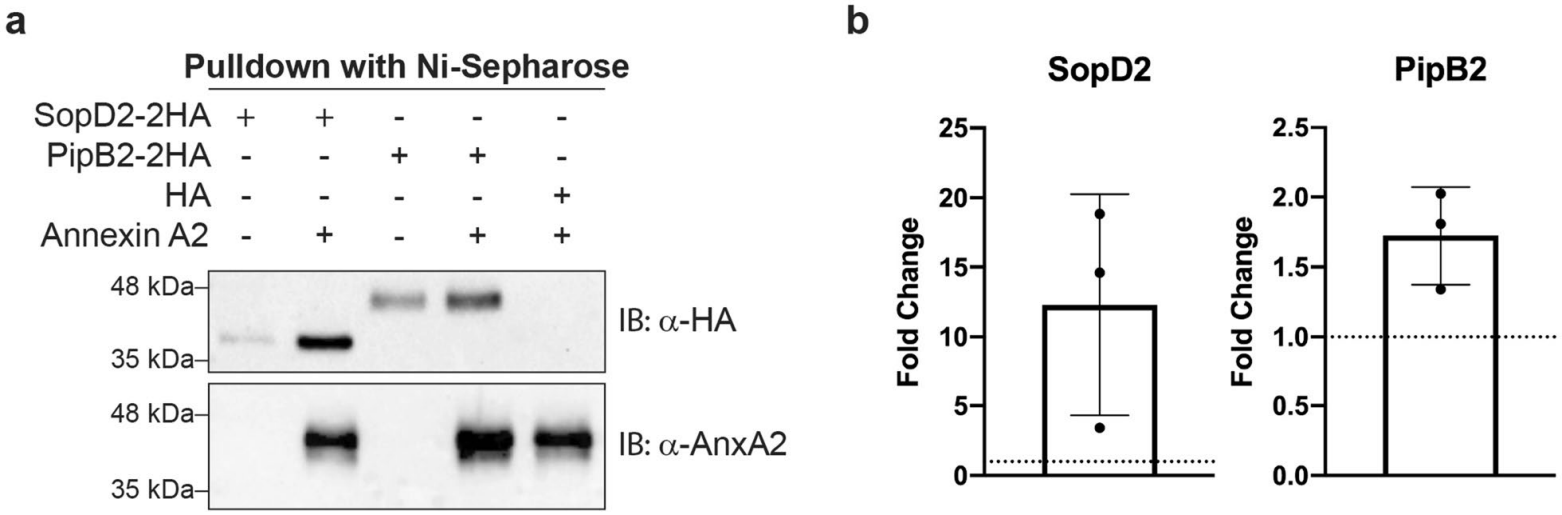
SopD2 targets AnxA2 in vitro. **(a)** Representative blot of reciprocal pull-down of AnxA2 and SopD2 or PipB2. Concentrated culture supernatants of *S.* Typhimurium strains secreting SopD2-2HA, PipB2-2HA, or HA empty vector were mixed with His_6_-AnxA2. Pull-downs were analyzed by Western blot using α-HA or α-AnxA2. IB: Immunoblot. Leftmost lane: pull-down of SopD2-2HA in the absence of AnxA2. Lane second from the left: pull-down of SopD2-2HA in the presence of AnxA2. Middle lane: pull-down of PipB2-2HA in the absence of AnxA2. Lane second from the right: pull-down of PipB2-2HA in the presence of AnxA2. Rightmost lane: pull-down of empty vector expressing a HA-tag in the presence of AnxA2. Original Western blots are shown in Supplemental Fig. S4. **(b)** Fold change determined by densitometry of 3 Western blots from independent experiments. Mean fold change ± standard deviation is shown. Fold change = (Densitometry of effector + AnxA2)/(Densitometry of effector). Dashed line = 1.

AnxA2 has previously been shown to be recruited to *S.* Typhimurium invasion sites in a T3SS1-dependent manner and is required for efficient invasion^48^. We decided to validate the interaction between AnxA2 and both SopD2 and PipB2 given that AnxA2 plays an important role in multiple steps of host cell membrane trafficking^49^. *S.* Typhimurium alters host endosome maturation in a T3SS2-dependent manner, and AnxA2 immunoprecipitated along with the T3SS2 effectors SopD2 and PipB2. To do so, we performed a reciprocal pull-down, using purified His_6_-tagged AnxA2 to enrich for SopD2-2HA and PipB2-2HA. Concentrated culture supernatant from T3SS2^+^*∆sopD2*-pSopD2-2HA, T3SS2^+^*∆pipB2*-pPipB2-2HA, and T3SS2^+^ -pACYC-2HA grown under SPI-2 inducing conditions was mixed with His_6_-tagged AnxA2, AnxA2 complexes were pulled-down against the His-tag, and analyzed by Western blotting. Both SopD2-2HA and PipB2-2HA were successfully pulled-down along with His_6_-AnxA2 (Fig. 5a).

SopD2 was pulled down substantially more in the presence of AnxA2 as compared to the pull-down in the absence of AnxA2 with an average fold change of 12.3 (Fig. 5b). This indicates that while SopD2-2HA can bind to Ni-Sepharose without AnxA2, the presence of AnxA2 considerably increases SopD2 pull down by 12.3-fold, strongly suggesting that SopD2 binds to AnxA2 during *S.* Typhimurium in vitro. Increased amounts of PipB2 were also pulled down in the presence of AnxA2 with an average fold change of 1.7. The apparent non-specific binding of PipB2-2HA (and SopD-2HA to a much lesser degree), to the Ni-Sepharose beads makes it difficult to conclude with certainty that PipB2 specifically binds to AnxA2, and alternative methods and future experiments might be needed to validate this interaction. However, in three independent experiments increased amounts of PipB2 were pulled down in the presence of AnxA2 as compared to in the absence of AnxA2 (Fig. 5b). The non-specific binding of SopD2-2HA and PipB2-2HA in the absence of AnxA2 could be due to exposed histidine residues on the outside surface of SopD2 and PipB2 resulting in effector pulldown by the Ni-Sepharose beads. In all, we can conclude that SopD2 targets AnxA2 and it appears likely from these data that PipB2 also directly interacts with AnxA2. It is also possible that AnxA2 is a component of a complex that interacts with SopD2 and PipB2 with stabilization provided by additional proteins such as plectin, vimentin, or alpha-actinin-4.

### Annexin A2 in *S.* Typhimurium infection

**Figure 6:**
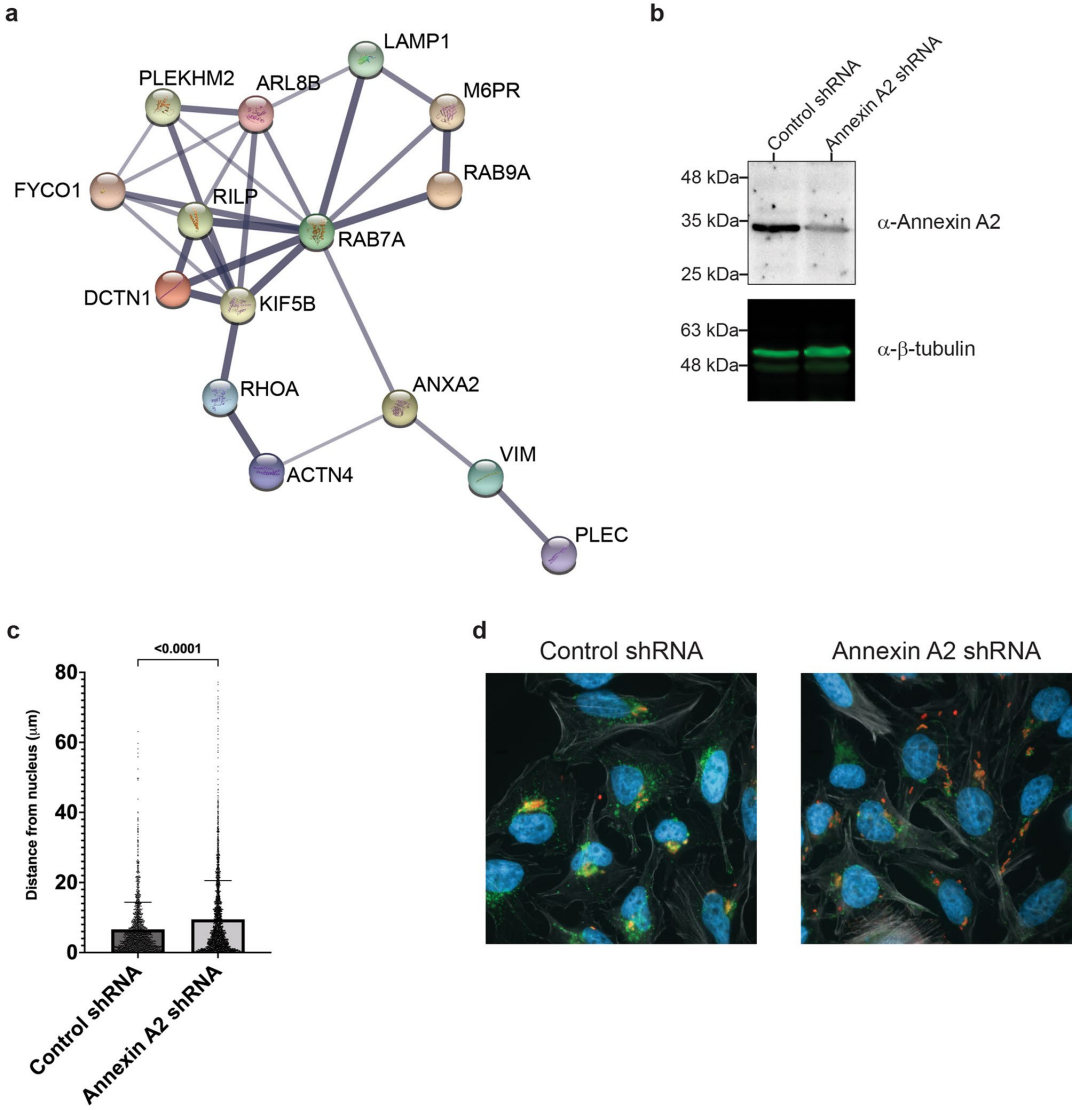
Annexin A2 contributes to the precise intracellular positioning of the SCV during infection. **(a)** STRING network showing both direct and indirect protein associations between AnxA2 and previously identified host proteins known to be involved in SIF biogenesis. Line thickness indicates the strength of data supporting the interaction with a minimum confidence of interaction score of 0.4. **(b)** Annexin A2 expression in Hela cells treated with either control shRNA (left lane) or with Annexin A2 shRNA (right lane) as determined by Western blot from whole cell lysate. Beta Tubulin was used as a loading control. Original Western blots are shown in Supplemental Fig. S5. **(c)** Distribution of *Salmonella* in infected HeLa cells treated with either control shRNA or Annexin A2 shRNA. shRNA-treated HeLa cells infected with wild type *Salmonella* were fixed at 8 h post infection, immunostained for *Salmonella* (red), LAMP1 (green), and actin (grey), and the nucleus was stained with DAPI (blue). The nearest edge distance from *Salmonella* to nucleus was measured in infected cells. The mean plus or minus SEM for three experiments is shown (*n* = 3). At least 200 distances per experiment were measured. The p-value is as indicated as determined by a Mann–Whitney test. **(d)** Select representative images of wild type *Salmonella*-infected HeLa cells treated with either control shRNA or Annexin A2 shRNA.

The process of SIF biogenesis requires the activity of several T3SS2-secreted effectors working in conjunction with each other in order to hijack the host cell’s endosomal system^3,50^. To our knowledge, AnxA2 has never been implicated in T3SS2-mediated processes. Given AnxA2’s variety of functions within the host cell including its involvement in the endocytic pathway, it is conceivable that AnxA2 could play a role in SIF biogenesis and SCV trafficking and intracellular positioning. To understand how AnxA2 could play a role in SIF biogenesis, we performed STRING analysis^47^ that included the four proteins previously identified by our SILAC screen (AnxA2, vimentin, plectin, and alpha-actinin-4) and other host proteins known to be involved in SIF biogenesis and SCV membrane maintenance identified in previous studies (Fig. 6).

The STRING analysis shown in Fig. 6a indicates that AnxA2 associates either directly (i.e., AnxA2 binds Rab7) or indirectly with Rab7, vimentin, and alpha-actinin-4. The interaction between AnxA2 and both vimentin and alpha-actinin 4, and tangentially plectin, highlights the role of AnxA2 in regulating cytoskeletal rearrangements within the host cell. Identification of vimentin and plectin in association with AnxA2 is likely due to AnxA2’s role within the host cell to regulate cytoskeletal rearrangements and may not be directly associated with T3SS2-secreted effectors. However, the association between AnxA2 and alpha-actinin 4, a F-actin crosslinking protein, may be linked to the T3SS2-mediated formation of an actin nest comprised of F-actin around the SCV several hours post invasion. There is also evidence that AnxA2 interacts either directly or indirectly with Rab7, a known target of SopD2. SopD2 modulates Rab7 activity thereby disrupting endocytic trafficking, and contributing to evasion of lysosomal degradation of intravacuolar *S.* Typhimurium^51^. SopD2 may bind both Rab7 and AnxA2 to alter the host’s endocytic maturation programme.

From these data, we theorize that SopD2, and likely PipB2, target AnxA2 to modulate formation of the actin nest around the SCV or participate in altering the host’s endosomal system to promote bacterial replication within the host cell. We first attempted immunostaining of AnxA2 and investigated its colocalization with SopD2-2HA and PipB2-2HA in *Salmonella*-infected HeLa cells (Supplemental Fig. S3c–f). AnxA2 staining displayed a scattered distribution pattern in the infected cells, and colocalized only occasionally with immunostained translocated SopD2-2HA and PipB2-2HA (Fig. S3e,f). It is possible that the nature of AnxA2 interaction with SopD2 and/or PipB2 is dynamic, and does not result in focusing or clustering of AnxA2 around the translocated effectors. To further assess AnxA2’s involvement in SopD2-mediated phenotypes during *Salmonella* infection, we did a knockdown of AnxA2 in HeLa cells using shRNA mediated RNA interference (Fig. 6b). Multiple T3SS2 effectors, including SopD2, are involved in the intracellular positioning of SCV near the nucleus and Golgi apparatus in infected epithelial cells. The *sopD2* deletion mutant has scattered distribution of SCVs throughout the host cell compared to wild type *S*. Typhimurium^11^. As shown in Fig. 6c,d, the SCV of wild type *S*. Typhimurium stayed in close proximity to the cell nucleus in HeLa cells transfected with scrambled control shRNA as expected, whereas it displayed a scattered distribution in the AnxA2 knockdown cells. This scattered SCV distribution pattern phenocopies the SCV distribution of the *sopD2* deletion mutant in HeLa cells with normal levels of AnxA2^11^. This strongly suggests that SopD2 interacts with AnxA2 to mediate its contribution in positioning the SCV.

## Discussion

As an intracellular pathogen, *S.* Typhimurium must enter host cells and establish an intracellular niche permissive of both survival and replication. *S.* Typhimurium relies on secretion of bacterial effectors into host cells to mediate both invasion and creation of its replicative niche. It is widely recognized that the T3SS2-secreted effectors are required to establish and maintain this intracellular niche; however, the precise mechanisms underpinning these processes remain ambiguous. Significant efforts over the years to identify T3SS2-secreted effector binding partners have provided many pieces to the puzzle but understanding how they fit together has proven challenging. Only in recent years have we begun to understand the function of SIFs during infection and small parts of the mechanism underpinning SIF biogenesis, but the mechanism as a whole remains unclear.

Recently, a study compared proximity-dependent biotin labelling (BioID) and immunoprecipitation coupled with mass spectrometry (IP-MS) to investigate T3SS2-secreted effectors known to manipulate host intracellular trafficking (SifA, SopD2, PipB2, SseF, and SseG)^40^. The authors found that the two methods identified different known interactors suggesting that BioID should be used to complement, rather than replace, traditional approaches. Furthermore, they suggested that the BioID data set produced by their work represents the molecular environment of the T3SS2 effectors and not necessarily effector binding partners^40^. Thus, we decided traditional techniques such as IP-MS better suits our purposes to identify direct effector binding partners and not proximal interactors.

A popular approach to studying T3SS2-effectors—namely SifA, SopD2, PipB2, SteA, SseJ, SseF, and SseG—is to transfect individual effectors into host cells and identify cognate binding partners^21,40,42,45,51–60^. This is the common approach owing to the relatively low abundance of T3SS2 effectors present within the host cell during infection. We demonstrated in a previous study that *S.* Typhimurium requires multiple effectors to establish its replicative niche within host cells, highlighting the need to study effectors as an ensemble rather than individually^11^. We hypothesize that multiple effectors may bind a single host target, either cooperating simultaneously or sequentially, to exert their function. Consequently, the absence of any single effector, whether through deletion or transfection of select effectors, may drastically impact identification of host binding partners. Thus, we created an isogenic *S.* Typhimurium strain that translocates an increased abundance of T3SS2 effectors into host cells during infection. To our knowledge, no one has attempted to identify multiple T3SS2-effector binding partners during infection while not heavily relying on transfection.

Here, we demonstrated that the T3SS2-secreted effector SopD2, and likely PipB2, target the host protein AnxA2. We first showed that SopD2-2HA and PipB2-2HA bind to AnxA2 by using SILAC-based IP-MS analysis of proteins from *Salmonella*-infected cells (Fig. 4), and then verified these interactions by in vitro pull-down assays (Fig. 5), although the non-specific binding of PipB2-2HA and, to a lesser extent SopD2-2HA, to the Ni-Sepharose beads makes it difficult to confirm this with absolute certainty. We also showed that AnxA2 plays an important role in the subcellular localization of the SCVs of wild type *Salmonella* infected HeLa cells. To our knowledge, this is the first report of a common binding partner for SopD2 and PipB2. AnxA2 is a pleiotropic phospholipid and Ca^2+^ binding protein that is expressed in a wide spectrum of cells and participates in many important host functions, including inflammation, autophagy, and even angiogenesis^61^. It has also been shown to regulate actin-associated processes at dynamic membranes including early and late endosomes^49,62^. AnxA2 plays an important role in host defense against some bacterial (such as *Klebsiella pneumoniae*) and fungal (such as *Cryptococcus neoformans*) infections, but may also be exploited for adhesion, invasion and proliferation by certain bacterial (such as *Pseudomonas aeruginosa* and *Rockettsia australis*) and viral (such as human papillomavirus) pathogens^61^. AnxA2 has previously been shown to be involved in T3SS1-mediated *Salmonella* invasion^48^, has only recently been shown to interact with SseI and SseL^63^ in macrophages, but has not been shown to be involved in T3SS2-mediated processes in epithelial cells. We hypothesize that AnxA2 could be involved in the T3SS2-mediated linkage between the SCV and the actin nest surrounding the SCV.

The single effector deletion mutants *∆sopD2* and *∆pipB2* have reduced replication within macrophages and also have altered subcellular localization relative to wild type infected cells^11,64,65^. These altered phenotypes in the *∆sopD2* and *∆pipB2* single mutants could be explained by altered interactions with the *S.* Typhimurium-induced vacuole-associated actin polymerization (VAP). The VAP plays an important role in SCV membrane integrity and may be involved in regulating the subcellular localization of the SCV during infection^66,67^. AnxA2 functions at sites of actin association with membranes enriched in cholesterol, such as the membrane of the SCV^49,68^. More specifically, AnxA2 is enriched at the junction between vesicles and actin, possibly functioning as a barbed-end capping protein^49^. Given that SopD2 and PipB2 bind AnxA2, we hypothesize that SopD2 and PipB2 recruit AnxA2 to the interface between the SCV and VAP to stabilize the F-actin filaments and link the SCV to the VAP. Deletion of SopD2 would lessen contact, or even completely detach the SCV from VAP, resulting in the observed scattered distribution of SCVs within the host cell cytoplasm as well as the decreased replication in macrophages for the *∆sopD2* mutant. Indeed, we show that RNAi knockdown of AnxA2 in HeLa cells alters intracellular positioning of SCV of wild type *S.* Typhimurium (Fig. 6c,d), which phenocopies the *sopD2* deletion mutant in HeLa cells with normal levels of AnxA2^11^. Our results suggest that SopD2 interacts with AnxA2 to modulate intracellular positioning of the SCV, but further experimentation will be needed to illuminate the mechanistic molecular details. It is also unclear how PipB2 would play into this connection between the SCV, AnxA2, and VAP, as the *∆pipB2* single effector deletion mutant remains in very close proximity to the Golgi during infection and the *∆pipB2* mutant only exhibits a mild decrease in intramacrophage replication relative to wild type^11,65^. Further research is required to determine whether AnxA2 is recruited to the VAP, whether this occurs in a T3SS2-dependant manner, and if binding of AnxA2 to either SopD2 or PipB2 contributes to VAP formation.

We were unable to identify any known interactors from previous studies in this work. However, it is important to note that our method differed in key ways from previous studies (reviewed in Ref.^10^). As previously mentioned, past studies relied heavily on transfection of single effectors into host cells to identify interacting proteins, discounting the possibility that two effectors are required for binding to the same host protein. Here, we infected host cells with *S.* Typhimurium with its entire T3SS2-secreted effector arsenal at its disposal, a strategy with its own advantages and disadvantages over transfection.

The biggest disadvantage to our technique is that the concentration of each effector within the host cell is low as compared to effectors introduced by transfection. Low effector concentration within the host cell makes it difficult to separate specific effector interactions from the background noise during mass spectrometry. We attempted to mitigate this limitation by creating and using the T3SS2^+^ *ssrB* overexpression strain which increases expression and secretion of T3SS2-secreted effectors. Nevertheless, the amount of tagged effector within the host cell is likely orders of magnitude less than that of a transfected effector. Protein complex formation is dependent on the stoichiometry of the interacting proteins. High protein concentrations resultant from effector transfection will shift the formation/dissociation equilibrium towards strong binding with one protein present at a greater molar ratio than the effector *e.g.*, the known interactors^69,70^. Given the low concentration of tagged effectors in our study, the formation/dissociation equilibrium is shifted toward what would be natively expected during normal cell infection, allowing for identification of different binding partners. This may explain why we did not identify known interactors.

The advantage of our native infection-based mass spectrometry study is that all effectors are present, permitting both effector-effector interactions and maintaining all effectors at biologically relevant levels, enabling identification of new effector binding partners. Effector transfection may introduce artificially high signal from the more highly expressed host proteins and drown out true effector binding partners. The absence of known binding partners in our study does not indicate a failed experiment, but rather highlights the differences in techniques used to identify host binding partners for T3SS2-secreted effectors.

Collectively, this study highlights the advantages of *S.* Typhimurium native infection in SILAC labelled cells as a valuable strategy to identify T3SS2-secreted effector binding partners. Using this technique, we demonstrate that SopD2 and PipB2 both bind to AnxA2 which may play a role in SCV intracellular positioning during infection. Further work is required to ascertain the precise role of AnxA2 during infection and the impact of AnxA2 binding to SopD2 and PipB2. Successful identification of a host protein associated with *S.* Typhimurium infection, and two T3SS2-effectors that bind that host protein, potentially explain the nuanced phenotypes observed in the single effector deletion mutants. Our work highlights that a global view of T3SS2-secreted effectors during infection is necessary to unravel the complexities of the *S.* Typhimurium intracellular replicative niche.

## Supplementary Information

Supplementary Information.
