# Supplementary material for: Quantitative proteomic screen identifies annexin A2 as a host target for Salmonella pathogenicity island-2 effectors SopD2 and PipB2
Source: Sci Rep. 2021 Dec 8;11:23630. doi: 10.1038/s41598-021-02795-x (PMC8655068; doi:10.1038/s41598-021-02795-x)
Supplement: Supplementary file 1 — Supplementary Information. [file 41598_2021_2795_MOESM1_ESM.pdf]

Quantitative proteomic screen identifies annexin A2 as a host target for *Salmonella* pathogenicity island-2 effectors SopD2 and PipB2

Katelyn Knuff-Janzen[1,2], Antonio Serapio-Palacios[1,2], James McCoy[1], Zakhar Krekhno[1,2], Kyung-Mee Moon[1,3], Wanyin Deng[1], Leonard J. Foster[1,3], B. Brett Finlay[1,2,3*]

[1] Michael Smith Laboratories, University of British Columbia, Vancouver, BC, Canada

[2] Department of Microbiology and Immunology, University of British Columbia, Vancouver, BC, Canada

[3] Department of Biochemistry and Molecular Biology, University of British Columbia, Vancouver, BC, Canada

[*] Corresponding author. Email: bfinlay@msl.ubc.ca; Phone +1-604-822-2210; Fax +1-604-822-9830

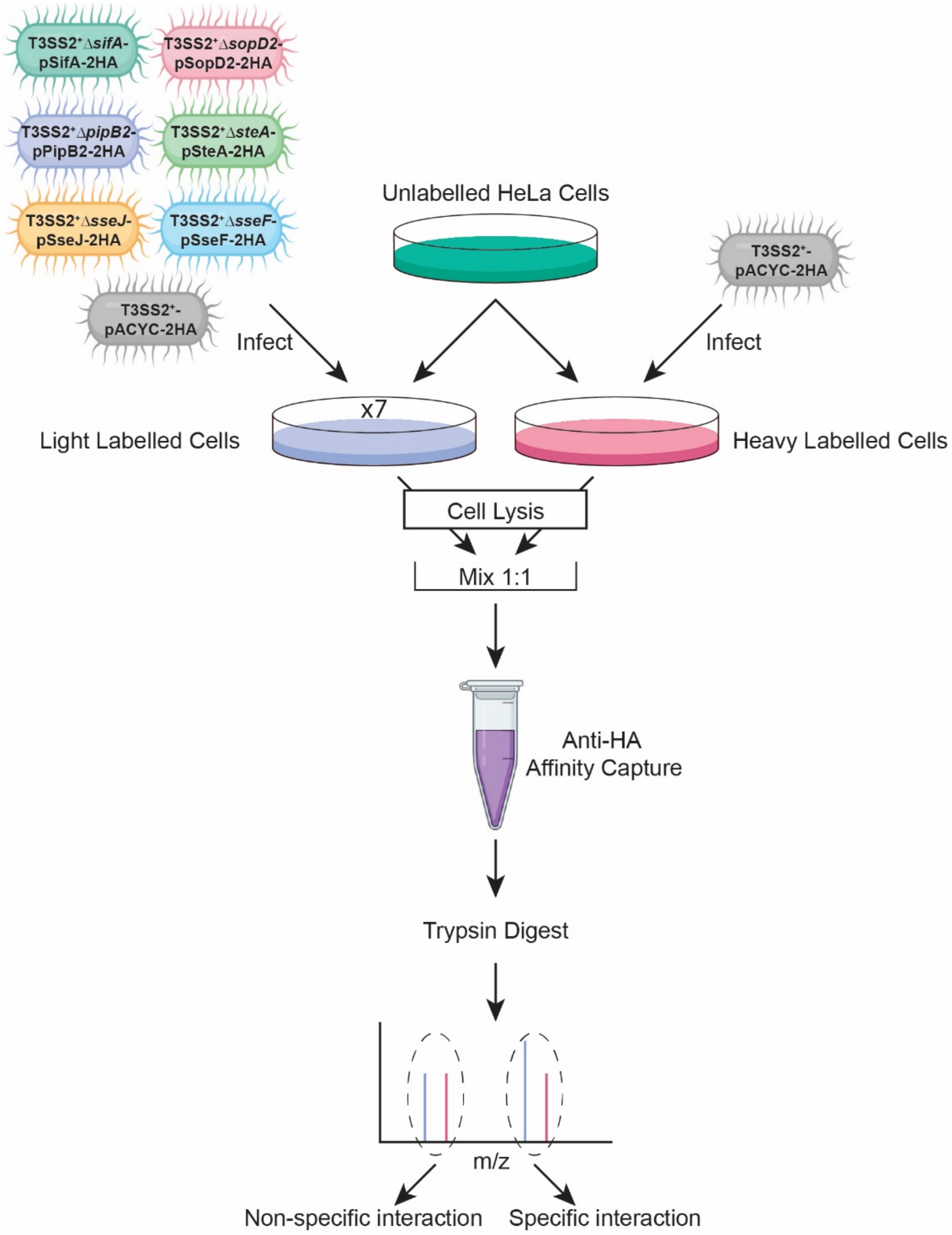

Supplemental Figure S1 – Experimental design. HeLa cells are grown in SILAC medium for at least 5 doublings to ensure complete incorporation of the isotope labels. Cells are infected with the indicated strains (one 15 cm dish for each strain) for 8 hours prior to cell lysis. Lysate from each dish of infected cells is mixed with an equivalent amount of lysate from heavy-labelled cells infected with a T3SS2$^+$ strain secreting the control vector. Samples are processed for simultaneous identification and quantification of host and bacterial peptides. Parts of figure were created using Biorender.com.

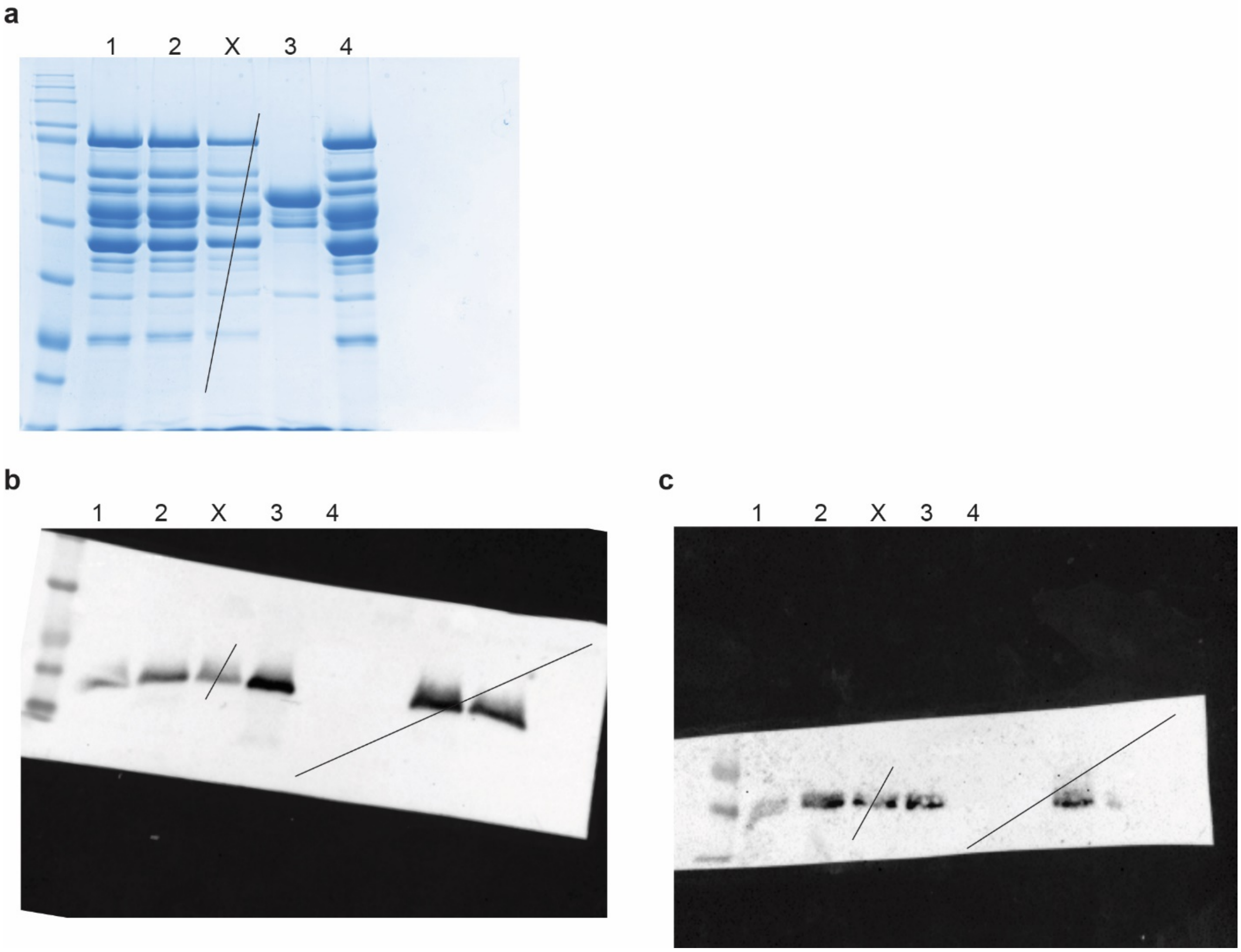

Supplemental Figure S2. (a) Original whole Coomassie-stained SDS-PAGE is shown. Gel imaging was performed zoomed in such that only the visible membrane edge is at the bottom. For the final figure, photoshop was used to remove the third lane (X) and the rest of the bands were placed in Figure 1a. **(b and c)**. Original western blots for Figure 1b and c, respectively, are shown. Blots were cut prior to antibody hybridization and imaging. For the final figure, photoshop was used to adjust the levels so that the bands were more apparent. The third lane (x) and the entire right side of each blot was removed, and the final images were placed in Figure 1 b and c, respectively.

a

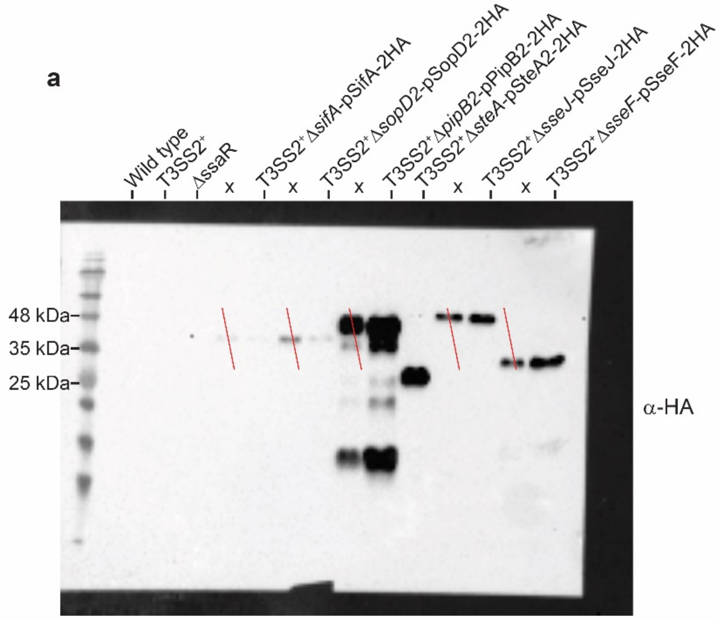
Wild type
T3SS2+
ΔssaR
x
T3SS2+ ΔsifA-pSifA-2HA
x
T3SS2+ ΔsopD2-pSopD2-2HA
x
T3SS2+ ΔpipB2-pPipB2-2HA
T3SS2+ ΔsteA-pSteA2-2HA
x
T3SS2+ ΔsseJ-pSseJ-2HA
x
T3SS2+ ΔsseF-pSseF-2HA
48 kDa
35 kDa
25 kDa
α-HA

b

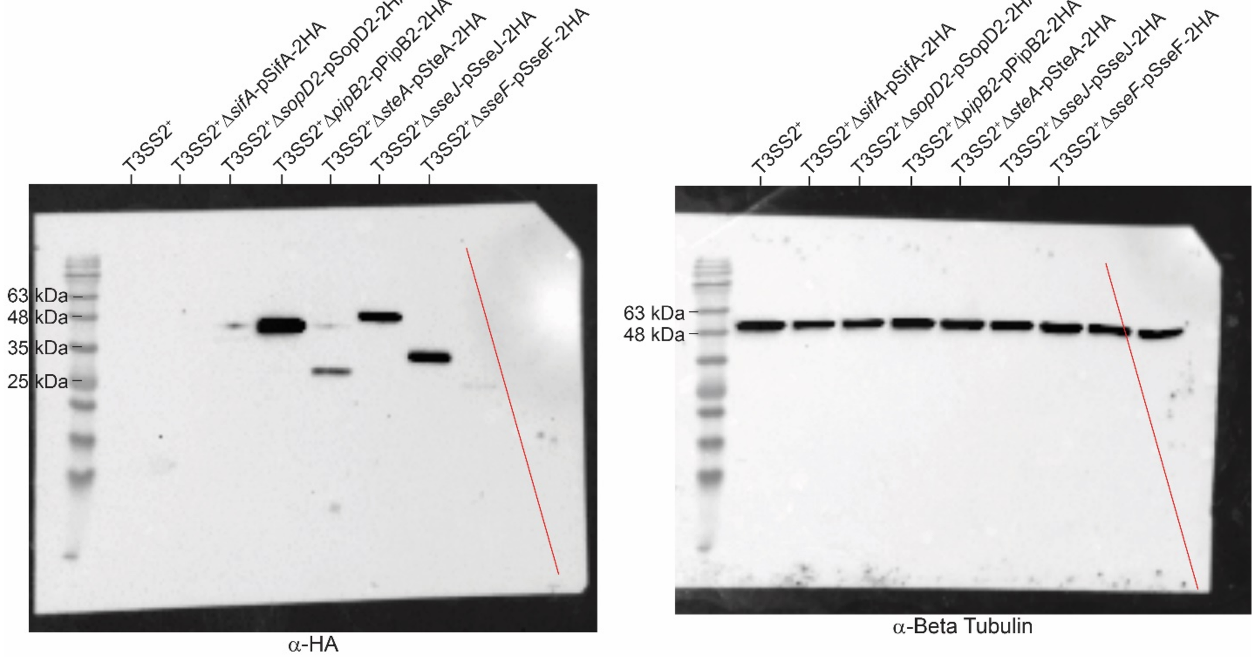
T3SS2+
T3SS2+ ΔsifA-pSifA-2HA
T3SS2+ ΔsopD2-pSopD2-2HA
T3SS2+ ΔpipB2-pPipB2-2HA
T3SS2+ ΔsteA-pSteA-2HA
T3SS2+ ΔsseJ-pSseJ-2HA
T3SS2+ ΔsseF-pSseF-2HA
63 kDa
48 kDa
35 kDa
25 kDa
α-HA
63 kDa
48 kDa
α-Beta Tubulin

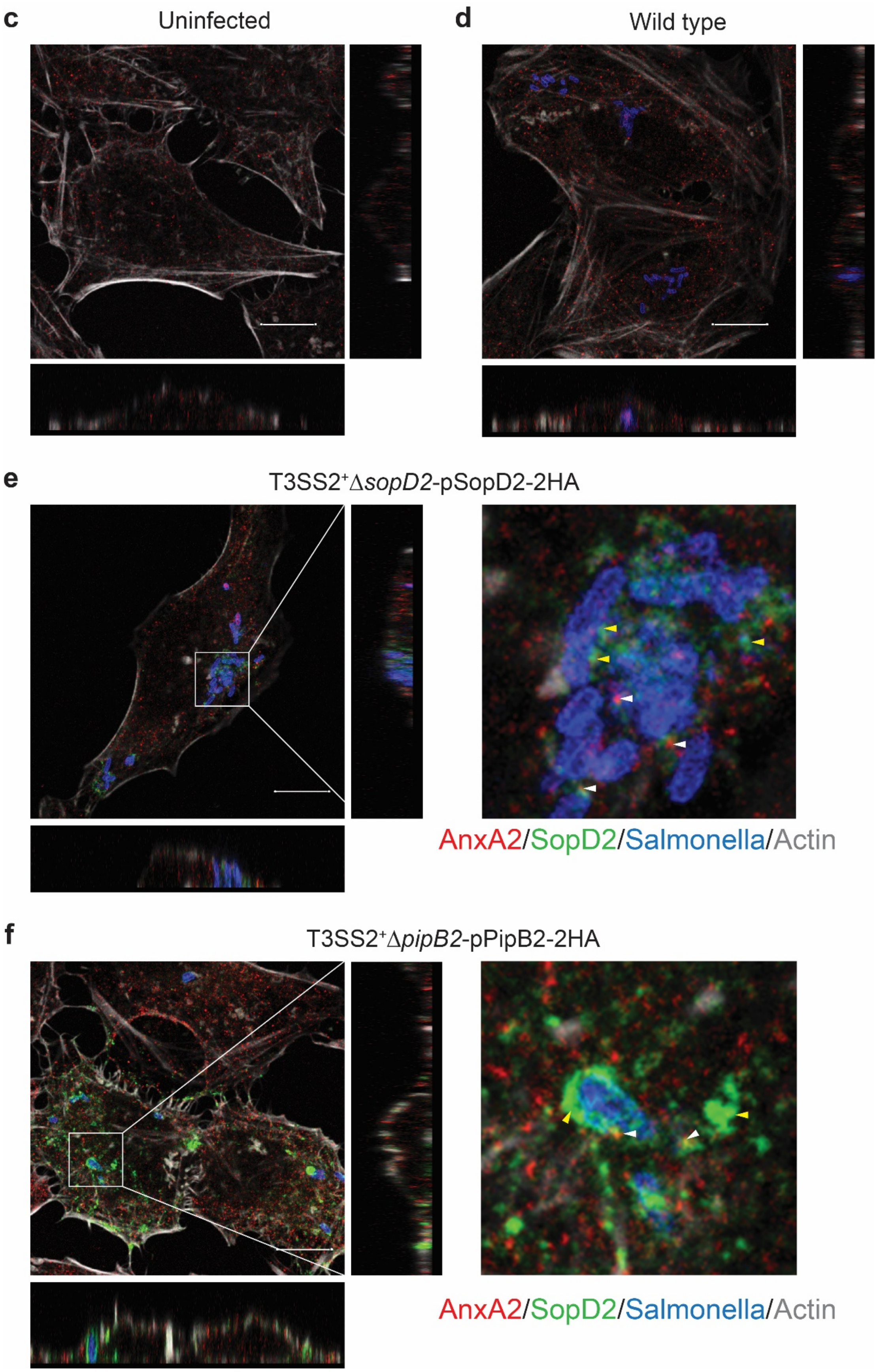
c
Uninfected
d
Wild type
e
T3SS2+ΔsopD2-pSopD2-2HA
AnxA2/SopD2/Salmonella/Actin
f
T3SS2+ΔpipB2-pPipB2-2HA
AnxA2/SopD2/Salmonella/Actin

Supplemental Figure S3. Tagged effectors are secreted *in vitro* and translocated during infection of HeLa cells. (a and b) Original Western blots are shown. **(a)** Blot is representative of *n*=3 experiments. Secreted proteins from indicated strains grown under SPI-2 inducing conditions were precipitated from sterilized culture supernatant and separated on an SDS-PAGE gel and immunoblotted with α-HA. **(b)** Tagged effectors are expressed during infection of HeLa cells. Blot is representative of *n*=3 experiments. Hela cells were infected with an MOI of 100 for each strain for 8 hours. Whole cell lysates were separated on an SDS 12% PAGE gel and immunoblotted with α-HA and α-Beta Tubulin (loading control) antibodies). **(c-f)** HeLa cells were infected at an MOI ≈ 100 with the indicated strains for 8 hours prior to cell fixation. Cells were immunostained for *Salmonella* (blue), HA (green), Annexin A2 (red), and actin (grey) and imaged by confocal microscopy. Orthogonal cross-sections are shown corresponding to one single slice of the Z-stack. **(e and f)** White boxes indicate zoomed-in region shown on the right. White arrowheads indicate Annexin A2 and yellow arrowheads indicate translocated SopD2-2HA and PipB2-2HA (**e** and **f**, respectively)

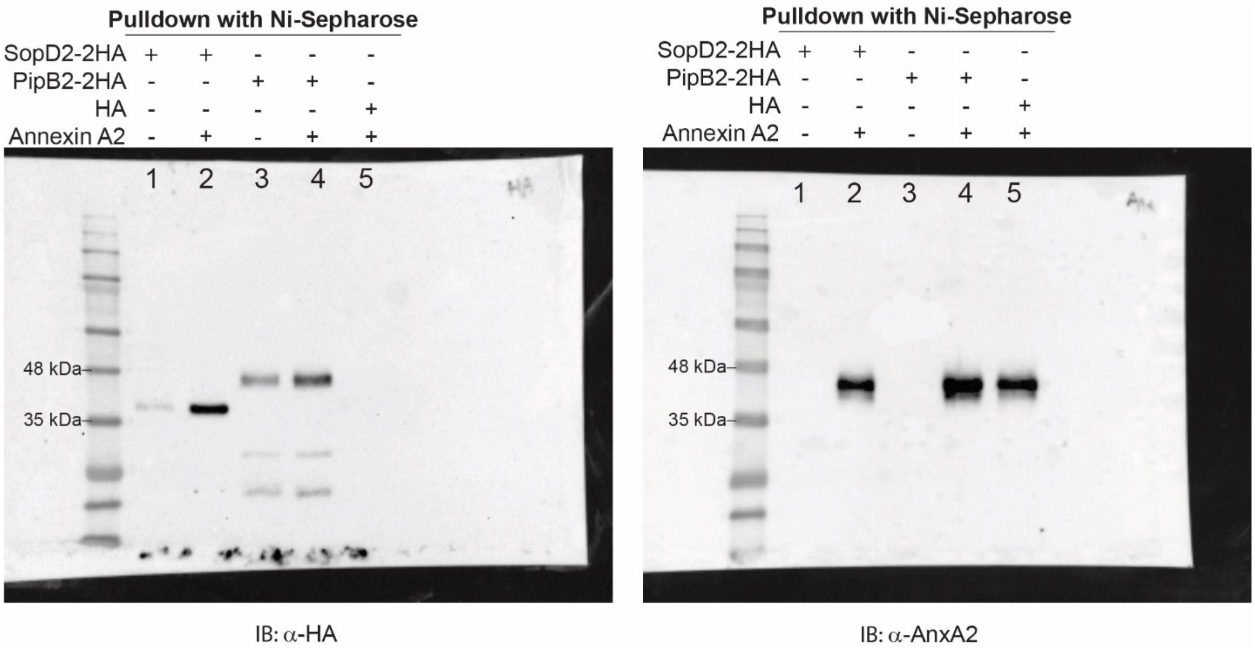

Supplementary Figure S4. Original Western blots are shown. For the final figure, Photoshop was used to remove everything to the right of Lane 5 and cropped to show just the bands of interest. The bands in lanes 1-5 were placed in Figure 5a.

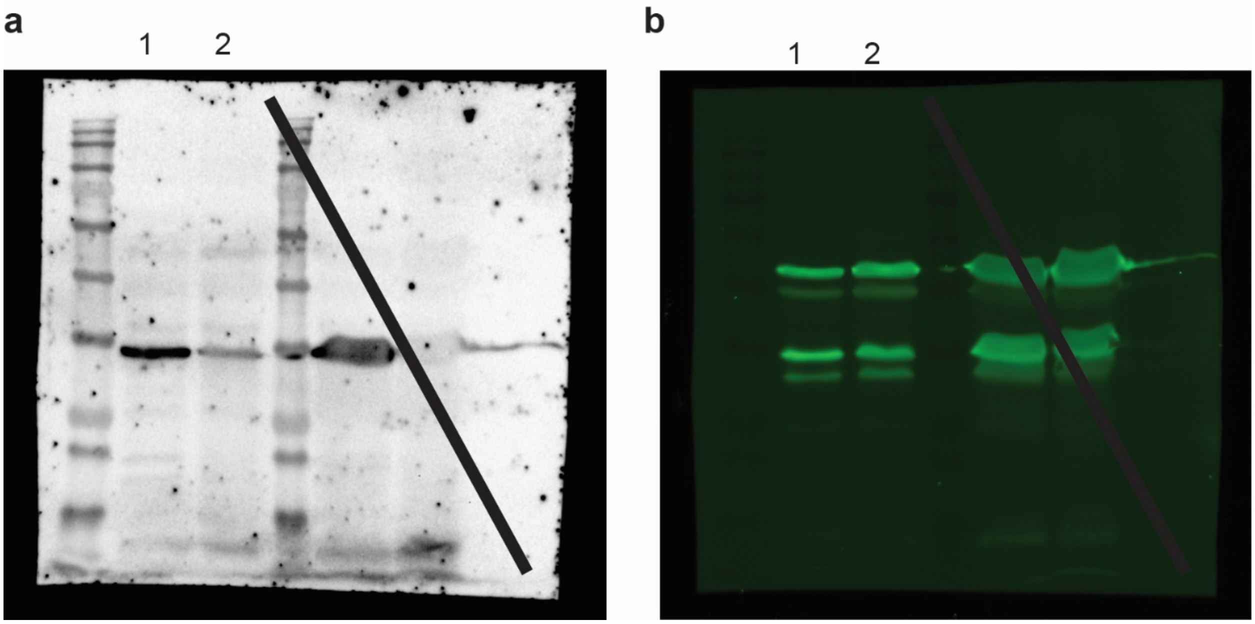

Supplementary Figure S5. (a and b) Original whole Western blots are shown. For the final figure, Photoshop was used to remove everything to the right of lane 2. The bands in lanes 1 and 2 were placed in Figure 6b.

Supplementary Dataset - The mass spectrometry proteomics data are available via ProteomeXchange.org with the identifier PXD025582. To access, use the following login credentials:

Username: reviewer_pxd025582@ebi.ac.uk

Password: qamwDKkH
